# Dopamine and Glutamate in Antipsychotic-Responsive Compared With Antipsychotic-Nonresponsive Psychosis: A Multicenter Positron Emission Tomography and Magnetic Resonance Spectroscopy Study (STRATA)

**DOI:** 10.1093/schbul/sbaa128

**Published:** 2020-09-10

**Authors:** Alice Egerton, Anna Murphy, Jacek Donocik, Adriana Anton, Gareth J Barker, Tracy Collier, Bill Deakin, Richard Drake, Emma Eliasson, Richard Emsley, Catherine J Gregory, Kira Griffiths, Shitij Kapur, Laura Kassoumeri, Laura Knight, Emily J B Lambe, Stephen M Lawrie, Jane Lees, Shôn Lewis, David J Lythgoe, Julian Matthews, Philip McGuire, Lily McNamee, Scott Semple, Alexander D Shaw, Krish D Singh, Charlotte Stockton-Powdrell, Peter S Talbot, Mattia Veronese, Ernest Wagner, James T R Walters, Stephen R Williams, James H MacCabe, Oliver D Howes

**Affiliations:** 1 Department of Psychosis Studies, Institute of Psychiatry, Psychology & Neuroscience, King’s College London, London, UK; 2 NIHR Biomedical Research Centre at South London and Maudsley NHS Foundation Trust, London, UK; 3 Division of Neuroscience and Experimental Psychology, School of Biological Sciences, Faculty of Biology, Medicine and Health, University of Manchester, Manchester, UK; 4 Academic Unit of Radiology, Medical School, Faculty of Medicine, Dentistry & Health, University of Sheffield, Sheffield, UK; 5 Department of Neuroimaging, Institute of Psychiatry, Psychology & Neuroscience, King’s College London, London, UK; 6 Division of Psychology and Mental Health, School of Biological Sciences, Faculty of Biology, Medicine and Health, University of Manchester, Manchester, UK; 7 Division of Psychiatry, University of Edinburgh, Edinburgh, UK; 8 Department of Biostatistics and Health Informatics, Institute of Psychiatry, Psychology & Neuroscience, King’s College London, London, UK; 9 Faculty of Medicine, Dentistry and Health Sciences, University of Melbourne, Parkville, Victoria, Australia; 10 CUBRIC, School of Psychology, College of Biomedical and Life Sciences, Cardiff University, Cardiff, UK; 11 Centre for Cardiovascular Science, University of Edinburgh, Edinburgh, UK; 12 MRC Centre for Neuropsychiatric Genetics and Genomics, Division of Psychological Medicine and Clinical Neurosciences, School of Medicine, Cardiff University, Cardiff, UK; 13 Division of Informatics, Imaging and Data Sciences, University of Manchester, Manchester, UK; 14 Psychiatric Imaging Group, MRC London Institute of Medical Sciences, Hammersmith Hospital, London, UK

**Keywords:** 1H-MRS, PET, antipsychotic response, treatment resistance, schizophrenia

## Abstract

The variability in the response to antipsychotic medication in schizophrenia may reflect between-patient differences in neurobiology. Recent cross-sectional neuroimaging studies suggest that a poorer therapeutic response is associated with relatively normal striatal dopamine synthesis capacity but elevated anterior cingulate cortex (ACC) glutamate levels. We sought to test whether these measures can differentiate patients with psychosis who are antipsychotic responsive from those who are antipsychotic nonresponsive in a multicenter cross-sectional study. ^1^H-magnetic resonance spectroscopy (^1^H-MRS) was used to measure glutamate levels (Glu_corr_) in the ACC and in the right striatum in 92 patients across 4 sites (48 responders [R] and 44 nonresponders [NR]). In 54 patients at 2 sites (25 R and 29 NR), we additionally acquired 3,4-dihydroxy-6-[18F]fluoro-l-phenylalanine (^18^F-DOPA) positron emission tomography (PET) to index striatal dopamine function (*K*_*i*_^cer^, min^−1^). The mean ACC Glu_corr_ was higher in the NR than the R group after adjustment for age and sex (*F*_1,80_ = 4.27; *P* = .04). This was associated with an area under the curve for the group discrimination of 0.59. There were no group differences in striatal dopamine function or striatal Glu_corr_. The results provide partial further support for a role of ACC glutamate, but not striatal dopamine synthesis, in determining the nature of the response to antipsychotic medication. The low discriminative accuracy might be improved in groups with greater clinical separation or increased in future studies that focus on the antipsychotic response at an earlier stage of the disorder and integrate other candidate predictive biomarkers. Greater harmonization of multicenter PET and ^1^H-MRS may also improve sensitivity.

## Introduction

The degree to which symptoms of schizophrenia will improve with antipsychotic medication is extremely variable. For some patients, antipsychotics can be very effective in improving symptoms. However, a majority of patients experience only a partial improvement,^1–3^ and around a third of all patients meet criteria for treatment-resistant schizophrenia (TRS), for which the only recommended antipsychotic is clozapine.^4–6^ The difficulty of identifying TRS by clinical criteria, combined with a reluctance to prescribe clozapine, leads to a delay in clozapine initiation during which time patients are exposed to ineffective medications and symptoms are active and disabling.^7^ There is an initial indication that delay in clozapine prescription is associated with a worse response when clozapine is eventually prescribed.^8^

Emerging biological and epidemiological evidence suggests that antipsychotic nonresponsive illness could be categorically distinct from antipsychotic responsive illness.^9–14^ Elucidating the pathophysiology of antipsychotic nonresponse could identify new targets for drug development and could also enable the development of predictive biomarkers to identify such patients early in the illness, allowing treatment with clozapine to begin earlier.

A prominent neurochemical hypothesis of schizophrenia centers on elevated dopamine synthesis and release in the striatum, arising from increased activity in mesostriatal dopamine neurons.^15^ The blockade of striatal D_2_ dopamine receptors is considered a critical feature of antipsychotic efficacy.^16^ While the response may require a threshold level of D_2_ occupancy, in antipsychotic nonresponsive schizophrenia, symptoms may persist despite high levels of D_2_ blockade.^17,18^ This raises the possibility that antipsychotic nonresponsive patients have a different pathophysiology that is not addressed by D_2_ blockade.^19^ Recently, molecular imaging studies have shown that striatal dopamine synthesis capacity is lower in TRS relative to that in patients who respond to antipsychotics.^20,21^ In longitudinal studies, higher levels of striatal D_2_ occupancy by dopamine^22,23^ and striatal dopamine synthesis capacity^24^ are associated with a greater response to antipsychotic treatment. Thus, biomarkers of striatal hyperdopaminergia may be predictive of an increased likelihood to respond to first-line (D_2_ blocking) antipsychotic treatment.

If TRS is not associated with abnormal striatal dopamine synthesis capacity, then the pathophysiology probably lies elsewhere. One possibility is that TRS arises due to abnormal glutamatergic signaling, particularly in cortical areas.^25^ A series of cross-sectional studies have indicated that poor antipsychotic response is associated with a higher level of glutamate metabolites in the anterior cingulate cortex (ACC)^14,26–28^ relative to levels in patients who have shown a good response or healthy volunteers. In first-episode psychosis, a higher level of ACC glutamate is predictive of a worse response to antipsychotic treatment.^29^ Higher frontal glutamate metabolites are also predictive of a poor response following reinitiation of antipsychotic treatment.^30^ In the striatum, glutamate metabolites may be elevated at illness onset^31,32^ but the relationship with the antipsychotic response is less clear.^27,28,33–35^ These observations may be particularly important in the context of the substantial efforts to develop glutamatergic drugs for schizophrenia, as they may suggest that glutamate modulation may be more effective in TRS than in antipsychotic-responsive patients.

So far, cross-sectional studies of dopaminergic^20–23^ or glutamatergic^14,26–28,33,36^ function in relation to antipsychotic response have been single-center studies that have recruited relatively small and homogenous patient cohorts. A key step in scaling this research toward developing predictive biomarkers for future stratified clinical trials is to test for these associations in a larger, more clinically representative patient sample and to determine the accuracy of group discrimination. The main aim of the current study was, therefore, to determine if glutamate levels in the ACC and striatum and striatal dopamine synthesis capacity differentiate antipsychotic nonresponsive from antipsychotic responsive psychosis in a multicenter cross-sectional sample. We hypothesized that, compared with the antipsychotic-responsive group, antipsychotic nonresponse would be characterized by lower striatal dopamine synthesis capacity and higher glutamate levels in the striatum and ACC. A secondary aim was to investigate relationships between ACC and striatal glutamate and striatal dopamine synthesis capacity in the same individuals.

## Methods

### Regulatory Approvals

The study had NHS Research Ethics Committee (15/LO/0038) and Administration of Radioactive Substances Advisory Committee (630/3764/32558) approvals. Participation required the provision of written informed consent.

### Participants

Study participants were recruited and assessed across 4 UK sites: King’s College London (KCL), University of Manchester (UoM), University of Edinburgh (UoE), and Cardiff University (CU). Inclusion criteria required that participants were aged between 18 and 65, met Diagnostic and Statistical Manual of Mental Disorders (DSM-5) criteria for schizophrenia or schizophreniform disorder, and were able to understand and consent to the study procedures. Exclusion criteria included currently meeting International Classification of Diseases (ICD) criteria for harmful substance misuse, or psychotic disorder secondary to substance misuse, pregnancy, previous severe head injury involving loss of consciousness for >5 minutes, and for Magnetic Resonance Imaging (MRI) presence of any contraindications to MRI at 3 tesla including implanted electronic devices or metallic objects. Treatment with clozapine in the last 3 months was an exclusion criterion, as the superior efficacy of clozapine in TRS^37^ could reflect differential biological effects. The cohort reflect a new patient sample, separate to those in our previous reports.^14,26,29,36,38^ Volunteers were reimbursed for participating in MRI and positron emission tomography (PET) scans.

The Mini International Neuropsychiatric Interview (MINI)^39^ was used to aid clinical diagnosis. Medication history and antipsychotic response were recorded through a structured interview and review of medical records. Antipsychotic doses were converted to chlorpromazine equivalent (CPZE) doses using the method of Davis and Chen,^40^ with the exception of amisulpride that used defined daily dose (https://www.whocc.no/atc_ddd_index/). Illness severity was evaluated using the Positive and Negative Syndrome Scale (PANSS)^41^ and the Clinical Global Impression scale for Schizophrenia (CGI-SCH).^42^

### Definition of Antipsychotic Responder and Antipsychotic Nonresponder groups

Antipsychotic Responders (R) were defined as having had (1) treatment with only 1 antipsychotic drug since illness onset, or, if there were any treatment changes, then these were due to adverse effects as opposed to nonresponse; (2) a CGI-SCH severity score of <4; (3) a PANSS total score of <60^43^; and (4) a compliance rating scale (CRS) score^44^ of >3.

Antipsychotic nonresponders (NR) were defined as having (1) documented treatment with at least 2 antipsychotics for >4 weeks each, at doses above the minimum therapeutic doses as defined by the British National Formulary; (2) a CGI-SCH severity score of >3; (3) a PANSS total score of at least 70; and (4) a CRS of >3. The targets for participant enrollment differed by site, but each site aimed to recruit a 1:1 ratio of R and NR.

### Proton Magnetic Resonance Spectroscopy (^1^H-Magnetic Resonance Spectroscopy)

Glutamate levels were measured using ^1^H-magnetic resonance spectroscopy (^1^H-MRS) at 3 tesla at all 4 sites (see supplementary Methods). Non-rotated ^1^H-MRS voxels were positioned in the ACC (20 × 20 × 20 mm^3^; supplementary figure 1)^26^ and in the right striatum (20 × 20 × 20 mm^3^; supplementary figure 2).^31^ Spectra were acquired using Point RESolved Spectroscopy (PRESS, echo time = 35 ms; repetition time = 2000 ms; 128 averages, bandwidth/sample frequency ±2500 Hz, complex points = 4096), and analyzed in LCModel version 6.3-1L^45^ using a standard LCModel basis set. Representative spectra are provided in supplementary figure 3. Metabolite estimates were water-referenced. Gannet software (version 2.0, http://www.gabamrs.com/) co-registered the ^1^H-MRS voxel to the corresponding T1-weighted image to determine the voxel tissue composition. Metabolite values were corrected for voxel tissue content using the formula:

$$\begin{array}{l} Mcorr\;=\;M*(WM+1.21*GM+1.55*CSF)\;/\;\left(WM+GM\right) \end{array}$$

where M is the uncorrected metabolite concentration, and WM, GM, and CSF indicate the percentages of tissue type in the voxel.^46,47^Further details are provided in the supplementary information. The primary outcome variable was Glu_corr._ For completeness, data for glutamate plus glutamine (Glx_corr_), are also presented.

Quality of ^1^H-MRS was determined by a review of LCModel estimates of spectral line width and signal-to-noise ratio. Spectra were excluded under any of the following criteria (1) absence of corresponding unsuppressed water acquisition; (2) compared with the overall mean for the voxel across all sites and participants, spectral line width was 2 standard deviations above; or (3) spectral signal-to-noise ratio was 2 standard deviations below. Individual metabolite concentration estimates associated with Cramér Rao lower bounds (CRLB) > 20% were excluded. We relied on these quality control procedures to identify and exclude any datasets potentially corrupted by motion or other artifacts.

### 3,4-Dihydroxy-6-[18F]Fluoro-l-Phenylalanine Positron Emission Tomography

Striatal dopamine function was measured using 3,4-dihydroxy-6-[18F]fluoro-l-phenylalanine (18F-DOPA) PET. The study acquired ^18^F-DOPA PET scans in participants who had also participated in ^1^H-MRS, at 2 sites (KCL and the UoM). To reduce the formation of radiolabeled 18F-DOPA metabolites,^48^ participants received carbidopa (150 mg) and entacapone (400 mg) orally 1 hour before 18F-DOPA imaging.^49^ Thirty seconds after the start of PET image acquisition, approximately 150 MBq of ^18^F-DOPA was administered by bolus intravenous injection. Emission data were acquired in list mode over the 95-minute period immediately post-injection.

Head movement was corrected for by frame-by-frame realignment using mutual information image registration.^50,51^ An ^18^F-DOPA template,^52^ together with a striatal atlas,^53^ and cerebellum^54^ were nonlinearly normalized to each PET summation image in Statistical Parametric Mapping version 12 (http//www.fil.ion.ucl.ac.uk/spm) running in Matlab 2015b (Mathworks Inc.). This process allows automatic placement of volumes of interest (VOI) on individual PET images. The rate constant for the uptake of ^18^F-DOPA in the striatum (K_i_^cer^ min^−1^) was calculated using graphical analysis adapted for a reference tissue input function, using the cerebellum as the reference region.^55,56^ We investigated K_i_^cer^ across the whole striatal VOI, and in associative, sensorimotor, and limbic functional subdivisions^53^ reflecting the topographical arrangement of corticostriatal projections.^57^ As previously,^20,24^ data are reported across both hemispheres and we did not predict laterality of effect. Supplementary figure 4 provides an example of 18F-DOPA PET K_i_^cer^ images.

### Statistical Analysis

Due to site effects (see supplementary information), ^1^H-MRS metabolite concentration estimates and 18F-DOPA K_i_^cer^ values were converted to *Z*-scores, calculated by subtracting the site mean from individual values, before dividing by the site standard deviation. Potential influences of age or sex^58–61^ on Glu_corr_ or ^18^F-DOPA K_i_^cer^ min^-1^ were determined by Pearson’s correlation coefficient and *t*-tests. For primary analyses, analysis of variance compared Glu_corr_ and 18F-DOPA K_i_^cer^ min^-1^ in the antipsychotic R and antipsychotic NR groups, using a threshold for statistical significance of *P* < .05 (uncorrected) in SPSS (version 23, IBM). Where the effects of age or sex were detected, these variables were added to the model. To evaluate the accuracy of ACC Glu_corr_ and striatal 18F-DOPA K_i_^cer^ min^-1^ values in distinguishing between antipsychotic R and NR groups, receiver-operating curves (ROC) were estimated, using Stata (SE, version 14). The secondary analysis investigated continuous relationships between Glu_corr_ and 18F-DOPA K_i_^cer^ min^-1^ and PANSS scores using Pearson’s correlation coefficient. Effects of Glu_corr_ on ^18^F-DOPA K_i_^cer^ by the group were analyzed using analysis of variance.

## Results

### Participant Characteristics

Ninety-two participants (antipsychotic R, *n* = 48; antipsychotic NR, *n* = 44) completed ^1^H-MRS imaging (table 1) and 54 (R, *n* = 25; NR, *n* = 29) completed 18F-DOPA PET imaging (table 2). The demographic and clinical characteristics of the ^1^H-MRS and PET samples by the site are provided in supplementary tables 1 and 2. For participants who completed both ^1^H-MRS and PET, the mean interval between scans was 24.74 ± 25.06 days (range 1–116 days).

**Table 1.** Clinical and Demographic Characteristics of the ^1^H-MRS Sample

	Antipsychotic Responder	Antipsychotic Nonresponder	*P*
Sample size	48	44	
Age (years)	29.9 ± 9.8	28.9 ± 7.5	.64
Sex male/female	41/7	36/8	.64
Ethnicity			.96
White	27	24	
Black	13	14	
Asian	4	3	
Other	4	3	
Subtype			.19
Psychosis unspecified	15	7	
Schizophrenia	33	35	
Delusional disorder	0	1	
Schizoaffective disorder	0	1	
Current antipsychotic			.60
Aripiprazole	11	8	
Olanzapine	15	7	
Risperidone	9	5	
Amisulpride	2	4	
Quetiapine	3	9	
Paliperidone	1	4	
Zuclopenthixol	2	1	
Flupentixol	1	1	
Haloperidol	1	0	
Combination	3	5	
CPZE mg/day	426.5 ± 241.5	515.9 ± 379.6	.18
Other CNS medications			
None	40	34	
Antidepressants	5	9	.15
Benzodiazepines	4	6	.73
Age onset	24.6 ± 6.8	23.8 ± 6.4	.56
Duration of illness	5.1 ± 7.9	5.1 ± 5.1	.99
Tobacco daily/less than daily/not at all	21/4/23	17/3/24	.59
Cannabis ever Y/N	34/14	37/7	.25
Cannabis current Y/N	7/41	5/39	.76
Prior substance use disorder Y/N	1/47	0/44	
PANSS positive	12.0 ± 3.1	22.5 ± 3.5	
PANSS negative	13.5 ± 3.3	20.9 ± 4.8	
PANSS general	27.2 ± 4.3	43.3 ± 5.3	
PANSS total	52.7 ± 6.7	86.7 ± 8.8	

*Note*: Data are expressed as mean ± standard deviation unless otherwise specified. ^1^H-MRS, ^1^H-magnetic resonance spectroscopy; CNS, central nervous system; CPZE, chlorpromazine equivalent dose; PANSS, Positive and Negative Syndrome Scale. Current cannabis use was defined as use within the last 7 days. *P* values relate to independent samples *t*-tests, Chi square, or Fisher’s exact test as appropriate. There were no significant group differences in clinical or demographic characteristics other than in PANSS scores.

**Table 2.** Clinical and Demographic Characteristics of the ^18^F-DOPA PET Sample

	Antipsychotic Responder	Antipsychotic Nonresponder	*P*
Sample size	25	29	
Age (years)	29.8 ± 9.6	30.0 ± 8.3	.86
Sex male/female	21/4	24/5	1.00
Ethnicity			.77
White	10	11	
Black	9	13	
Asian	3	3	
Other	3	2	
Subtype			.23
Psychosis unspecified	8	6	
Schizophrenia	17	21	
Delusional disorder	0	1	
Schizoaffective disorder	0	1	
Current antipsychotic	7	6	.53
Aripiprazole	7	4	
Olanzapine Risperidone	5	2	
Amisulpride	2	3	
Quetiapine	1	4	
Paliperidone	0	4	
Clopixol	1	1	
Flupenthixol	1	1	
Haloperidol	1	0	
Combination	0	4	
CPZE (mg/day)	404.8 ± 224.6	557.8 ± 420.9	.11
Other CNS medications			
None	21	23	
Antidepressants	4	5	1.00
Benzodiazepines	1	3	.62
Age onset	23.4 ± 5.5	24.1 ± 6.9	.72
Duration of illness	5.9 ± 9.1	5.9 ± 5.7	.98
Tobacco daily/less than daily/not at all	10/2/13	10/1/18	.57
Cannabis ever Y/N	17/8	23/6	.37
Cannabis current Y/N	3/22	3/26	1.00
PANSS positive	12.8 ± 3.2	22.0 ± 3.7	
PANSS negative	13.3 ± 3.1	22.3 ± 4.4	
PANSS general	27.0 ± 2.9	42.6 ± 5.1	
PANSS total	53.1 ± 5.5	86.9 ± 9.5	
^18^F-DOPA dose MBq	156.06 ± 15.93	155.01 ± 14.89	.81

*Note*: Data are expressed as mean ± standard deviation unless otherwise specified. ^18^F-DOPA PET, 3,4-dihydroxy-6-[18F]fluoro-l-phenylalanine positron emission tomography; CNS, central nervous system; CPZE, chlorpromazine equivalent dose; PANSS, Positive and Negative Syndrome Scale. Current cannabis use was defined as use within the last 7 days. *P* values relate to independent samples *t*-tests, Chi square, or Fisher’s exact test as appropriate. There were no significant group differences in clinical or demographic characteristics other than in PANSS scores.

### Glutamate Metabolite Levels

**Fig. 1.**
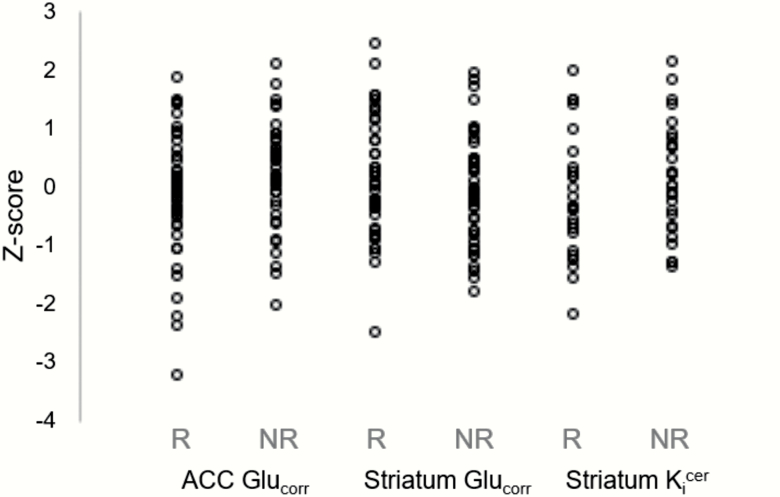
Glutamate and dopamine measures in the antipsychotic responder and antipsychotic nonresponder groups. Glutamate is expressed as the corrected ^1^H-MRS glutamate concentration (Glu_corr_) in the anterior cingulate cortex (ACC) and right striatum. Dopamine synthesis was measured as ^18^F-DOPA *K*_i_^cer^ min^−1^ across the whole striatum. Values are presented as *Z*-scores. Data are shown in antipsychotic responder (R) and antipsychotic nonresponder (NR) groups.

**Fig. 2.**
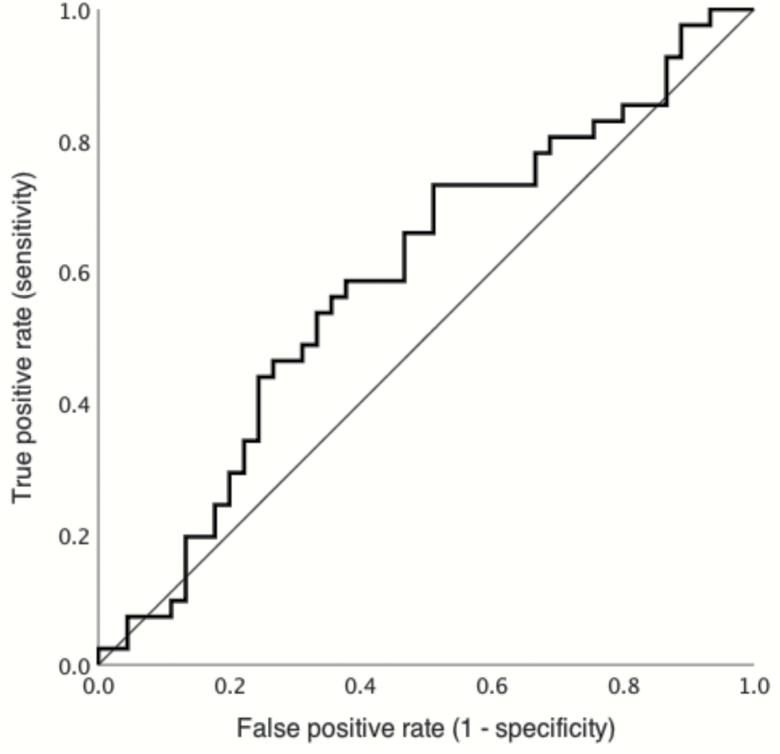
Receiver operating characteristic (ROC) curve of the ability for glutamate (Glu_corr_) in the anterior cingulate cortex (ACC) to discriminate the antipsychotic responder (R) and antipsychotic nonresponder (NR) groups. The area under curve (AUC) is 0.59. The straight line represents chance performance.

ACC Glu_corr_ and Glx_corr_ were related to age (*N* = 86; Glu_corr_*r =* −.21; *P* = .05, Glx_corr_*r* = −.27; *P* = .01; supplementary figure 4), and sex (mean ± s.d. Glu_corr_ male: 0.10 ± 0.94; female: −0.52 ± 1.04; *T*_84_ = 2.21; *P* = .03; Glx_corr_ male: 0.11 ± 0.93; female: −0.56 ± 1.09; *T*_84_ = 2.40; *P* = .01). There were no significant effects of current tobacco or cannabis use or antipsychotic CPZE dose (*P* > .14). The NR group had significantly higher ACC Glu_corr_ levels compared with the R group after adjustment for age and sex (main effect of group: Glu_corr_*F*_1,81_ = 4.99; *P* = .03; η ^2^ = 0.06; table 3, figure 1). A similar result at threshold levels of significance was detected for Glx_corr_ (*F*_1,81_ = 3.92; *P* = .05; η ^2^ = 0.05; table 3, figure 1). Interactions between group and age or group and sex did not show any evidence of an effect. After excluding participants who were currently taking benzodiazepines (*n* = 10) or antidepressants (*n* = 14), the effects of group on Glu_corr_ remained borderline significant (*P* = .04 and *P* = .07, respectively). The effect of group was not significant in unadjusted analysis (Glu_corr_*F*_1,84_ = 2.37; *P* = .13; Glx_corr_*F*_1,84_ = 1.77; *P* = .19; table 3). ROC analysis of non-adjusted ACC Glu_corr_ levels in the R and NR groups returned an area under curve (AUC) of 0.59 (figure 2). Subsequent empirical cut-point estimation returned an optimal cut point of 0.98, which was associated with a Youden index of 0.22, sensitivity = 0.73, specificity = 0.49, and AUC of 0.61. There were no significant correlations between ACC Glu_corr_ and PANSS scores or CPZE dose (*N* = 86; *r* = −.04 to .15).

**Table 3.** Glutamate and Dopamine Measures in the Antipsychotic Responder and Antipsychotic Nonresponder Groups

	Antipsychotic Responder	Antipsychotic Nonresponder	ES, or GLM, Group	GLM: Group, Age, and Sex
^1^H-MRS glutamate (Glu_corr_)				
Anterior cingulate cortex				
KCL	19.39 ± 3.56 (16)	20.29 ± 2.63 (18)	*d* = 0.29	
UoM	13.74 ± 1.75 (17)	14.28 ± 1.59 (15)	*d* = 0.74	
UoE	12.57 ± 1.18 (7)	13.33 ± 0.90 (5)	*d* = 0.72	
CU	11.65 ± 1.68 (5)	11.57 ± 2.22 (3)	*d* = 0.04	
Overall	−0.15 ± 1.05 (45)	0.17 ± 0.88 (41)	*F*_1,84_ = 2.37; *P* = .13; η ^2^ = 0.03	*F*_1,81_ = 4.99; *P* = .03; η ^2^ = 0.06
Right striatum				
KCL	10.78 ± 1.47 (16)	11.07 ± 1.62 (18)	*d* = 0.19	
UoM	8.35 ± 1.12 (17)	7.89 ± 1.01 (15)	*d* = 0.43	
UoE	8.67 ± 1.13 (5)	7.41 ± 1.21 (4)	*d* =1.08	
CU	9.00 ± 3.15 (4)	8.58 ± 1.43 (4)	*d* = 0.17	
Overall	0.10 ± 1.00 (42)	−0.11 ± 0.96 (41)	*F*_1,81_ = 0.96; *P* = .33; η ^2^ = 0.12	
^1^H-MRS Glx (Glx_corr_)				
Anterior cingulate cortex				
KCL	25.86 ± 5.28 (16)	26.85 ± 4.28 (18)	*d* = 0.21	
UoM	19.67 ± 2.56 (17)	20.17 ± 1.87 (15)	*d* = 0.22	
UoE	18.59 ± 1.61 (7)	19.62 ± 1.87 (5)	*d* = 0.59	
CU	14.82 ± 2.15 (5)	15.56 ± 2.14 (3)	*d* = 0.34	
Overall	−0.13 ± 1.06 (45)	0.15 ± 0.88 (41)	*F*_1,84_ = 1.77; *P* = .19; η ^2^ = 0.02	*F*_1,81_ = 3.92; *P* = .05; η ^2^ = 0.05
Right striatum				
KCL	15.08 ± 2.65 (16)	14.27 ± 3.15 (18)	*d* = 0.29	
UoM	13.34 ± 1.81 (17)	12.89 ± 2.33 (15)	*d* = 0.22	
UoE	16.69 ± 5.84 (5)	12.70 ± 3.20 (4)	*d* = 0.85	
CU	11.75 ± 3.53 (4)	14.10 ± 3.41 (3)	*d* = 0.68	
Overall	0.13 ± 0.91 (42)	−0.13 ± 1.04 (40)	*F*_1,81_ = 1.39; *P* = .24; η ^2^ = 0.01	
^18^F-DOPA PET K_i_^cer^				
Whole striatum				
KCL	0.0125 ± 0.0095 (11)	0.0128 ± 0.0010 (16)	*d* = 0.04	
UoM	0.0139 ± 0.0013 (14)	0.0143 ± 0.0010 (13)	*d* = 0.34	
Overall	−0.18 ± 1.05 (25)	0.12 ± 0.93 (29)	*F*_1,52_ = 1.24; *P* = .27; η ^2^ = 0.02	
Sensorimotor striatum				
KCL	0.0126 ± 0.0011 (11)	0.0149 ± 0.0010 (16)	*d* = 2.19	
UoM	0.0149 ± 0.0016 (14)	0.0156 ± 0.0010 (13)	*d* = 0.52	
Overall	−0.25 ± 0.96 (25)	0.16 ± 0.95 (29)	*F*_1,52_ = 2.54; *P* = .12; η ^2^ = 0.05	
Associative striatum				
KCL	0.0126 ± 0.0010 (11)	0.0128 ± 0.0010 (16)	*d* = 0.2	
UoM	0.0136 ± 0.0014 (14)	0.0138 ± 0.0010 (13)	*d* =0.16	
Overall	−0.13 ± 1.07 (25)	0.09 ± 0.95 (29)	*F*_1,52_ = 0.62; *P* = .44; η ^2^ = 0.01	
Limbic striatum				
KCL	0.0122 ± 0.0010 (11)	0.0128 ± 0.0010 (16)	*d* = 0.6	
UoM	0.0136 ± 0.0012 (14)	0.0139 ± 0.0010 (13)	*d* = 0.3	
Overall	−0.14 ± 1.05 (25)	0.12 ± 0.99 (29)	*F*_1,52_ = 0.86; *P* = .36; η ^2^ = 0.02	

*Note*: Data are presented by site and as overall *Z*-score. Data are expressed as mean ± standard deviation (number of observations). ES, effect size; GLM, general linear model; ^1^H-MRS, ^1^H-magnetic resonance spectroscopy; ^18^F-DOPA PET, 3,4-dihydroxy-6-[18F]fluoro-l-phenylalanine positron emission tomography; KCL, King’s College London; UoM, University of Manchester; UoE, University of Edinburgh; CU, Cardiff University.

There was no association between striatal Glu_corr_ or Glx_corr_ and age (*N* = 83; Glu_corr_*r* = −.13, *P* = .25; Glx_corr_*r* = −.09, *P* = .44), or sex (Glu_corr_*T*_81_ = 0.30; *P* = .77; Glx_corr:_*T*_81_ = 1.76; *P* = .08). There were also no significant effects of current tobacco or cannabis use or antipsychotic CPZE dose (*P* > .07). There was no between-group difference in striatal Glu_corr_ (*F*_1,81_ = 0.96; *P* = .33; figure 1) or Glx_corr_ (*F*_1,81_ = 1.39; *P* = .24; table 3), or significant correlations between striatal Glu_corr_ or Glx_corr_ and PANSS scores or CPZE dose (*N* = 83; *r* = −.17 to .11). Site differences were present across ^1^H-MRS data (supplementary tables 3 and 4; supplementary Results).

### Striatal Dopamine Function

Striatal 18F-DOPA *K*_i_^cer^ values were not associated with age (*N* = 54; *r* = .07; *P* = .61), sex (*T*_52_ = 0.66; *P* = .51), tobacco (*F*_2,52_ = .17; *P* = .85), cannabis use (*F*_1,52_ = 1.20; *P* = .28), or antipsychotic CPZE dose (*r* = .06; *P* = .67). *K*_i_^cer^ did not differ between the R and NR groups (*F*_1,52_ = 1.24; *P* = .27; table 3, figure 1). ROC analysis of whole striatal 18F-DOPA *K*_i_^cer^ in antipsychotic R and NR returned an AUC of 0.59. *K*_i_^cer^ values were not associated with PANSS scores or CPZE dose (*r* = −.01 to .13).

### Relationships Between Glutamate and Dopamine

There was no main effect of ACC Glu_corr_ on striatal 18F-DOPA *K*_i_^cer^ (*F*_1,50_ = 1.03; *P* = .31), but the interaction between ACC Glu_corr_ and group was significant (*F*_1,50_ = 6.53; *P* = .01). This was related to a positive relationship between ACC Glu_corr_ and striatal 18F-DOPA *K*_i_^cer^ in NR (*N* = 29; *r* = .37; *P* = .05) but not in R (*N* = 25; *r* = −.31; *P* = .13). Striatal Glu_corr_ was negatively associated with striatal 18F-DOPA *K*_i_^cer^ across the whole sample (*F*_1,50_ = 4.97; *P* = .03), and there was no group by striatal Glu_corr_ interaction (*F*_1,50_ = 2.02; *P* = .16).

## Discussion

The main aim of this study was to test whether measures of dopamine synthesis capacity in the striatum^20,21^ and glutamate concentration (Glu_corr_) in the ACC^14,26^ could differentiate patients with antipsychotic-nonresponsive from antipsychotic-responsive schizophrenia. In line with our hypothesis, we found that ACC mean Glu_corr_ was higher in the NR compared with the R group, which was significant when age and sex were included in the model. There were no between-group differences in striatal dopamine function nor in striatal Glu_corr_. These results are partially consistent with previous evidence that the degree of antipsychotic response in schizophrenia may be related to ACC glutamate concentration^14,26–30^ but not with evidence linking response to striatal dopamine function.^20–24^ The AUC for both glutamate and dopamine measures indicated low discriminative accuracy. This indicates that these measures alone are unlikely to be sufficiently sensitive to identify chronic patients with antipsychotic nonresponsive from responsive illness in routine clinical practice.

The higher mean ACC Glu_corr_ in NR is broadly consistent with cross-sectional^14,26–28^ and prospective studies^29,30^ associating higher levels of ACC glutamatergic metabolites with a poor antipsychotic response. However, these studies differ in the glutamate measurement (glutamate or Glx) or Glu ratios (to creatine), sometimes corrected for voxel tissue composition. In addition, 2 studies did not detect differences in ACC glutamate metabolites between a TRS and antipsychotic R group,^28,33^ and 1 found ACC Glx, but not glutamate, was elevated in patients with ultra-resistant schizophrenia (URS) compared with healthy volunteers, but not in TRS or URS compared with antipsychotic responders.^27^ Together with the current findings, the overall literature not only may indicate an association between elevated ACC glutamatergic metabolites and antipsychotic nonresponse but also suggests that effect sizes may be small and influenced by methodological factors and sample characteristics. In terms of biological mechanism, one explanation is that patients who are less likely to respond to treatment exhibit greater elevations in frontal glutamate metabolites, potentially linked to a greater degree of N-methyl-D-aspartate (NMDA) receptor or gamma-aminobutyric-acid (GABA)ergic dysfunction resulting from genetic or developmental mechanisms. In addition, antipsychotic medication could have less impact on frontal glutamatergic dysfunction in those who respond poorly to treatment. In the striatum, the lack of group difference in Glu_corr_ is consistent with 2 recent cross-sectional studies examining TRS to first-line antipsychotic responders or healthy volunteers.^27,28^ This could indicate that elevations in striatal glutamate at illness onset^31,32^ are reduced during antipsychotic treatment^34^ irrespective of the response category. Alternatively, as we also observed no group difference in striatal 18F-DOPA *K*_i_^cer^, these findings may indicate that the participants selected for our samples did not markedly differ in the overall striatal pathophysiology.

In the 54 patients with dopamine measures evaluated across 2 sites, there was no group difference in striatal 18F-DOPA *K*_i_^cer^, indicating similar levels of presynaptic dopamine synthesis and storage capacity. This finding differs from previous smaller studies that have associated increased striatal dopamine function with a good antipsychotic response.^20–24^ Using the same 18F-DOPA PET method, we reported lower striatal *K*_i_^cer^ in 12 patients with TRS compared with 12 antipsychotic responders.^20^ In another study comparing a TRS group currently taking clozapine with antipsychotic-responsive patients, the resistant group again had lower *K*_i_^cer^ than the responders.^21^ In first-episode psychosis, striatal *K*_i_^cer^ was positively related to subsequent antipsychotic response.^24^ Lower availability of D_2_ receptors for radiotracer binding, which may reflect increased D_2_ occupancy by dopamine, was also associated with subsequent response to 6 weeks of treatment with amisulpride.^23^ A dopamine depletion study also indicated that higher levels of synaptic dopamine are predictive of a good antipsychotic response.^22^

Within cortico-striatal networks, counterbalancing pathways and feedback loops regulate neurotransmitter balance.^62^ For example, glutamate release can both increase and decrease dopamine levels,^63^ dopamine receptor activation modulates glutamate release,^64^ and dopamine neurons may co-release glutamate.^65^ In NR only, ACC Glu_corr_ was positively correlated to striatal 18F-DOPA *K*_i_^cer^. In contrast, striatal Glu_corr_ and striatal *K*_i_^cer^ were negatively correlated across the whole sample. We previously found that ACC glutamate and striatal *K*_i_^cer^ were negatively correlated in patients with early psychosis and no significant relationship between these variables in healthy controls.^66^ Correlations between striatal glutamate and striatal *K*_i_^cer^ have not previously been investigated in patients but are positively correlated in healthy volunteers.^67^ Together these findings could suggest that glutamate-dopamine relationships may change with illness onset, progression, or antipsychotic response. One potential mechanism may involve alterations in the balance between the opposing influences of direct and indirect glutamatergic projections from the cortex to mesostriatal dopamine neurons. This interpretation could be further examined in animal models and in longitudinal patient studies over the course of antipsychotic treatment.

Relative to previous research, a strength of the current study is the large sample size, which reduces the risk of false-positive findings. There are also design differences compared with previous studies that may contribute to the lack of group difference in dopamine measures and the marginal group difference in ACC glutamate measures. The criteria used to define the antipsychotic NR and R groups may have led to less clinical separation of these groups than in our previous 18F-DOPA PET^20^ and ^1^H-MRS^14^ studies. In the current study, the R group criteria allowed a higher level of symptom severity, while the NR group was less symptomatic and met fewer of the criteria for establishing treatment resistance.^68^ In addition, although the 2 groups were relatively well-matched for medication, we did not confirm adherence by measuring blood plasma antipsychotic levels. It is thus possible that the NR group may have included some participants whose symptoms were high because of partial nonadherence, rather than because they were “true” nonresponders.^69^

Further strengths of our study include the establishment of collaborative multicenter ^1^H-MRS and PET imaging in the UK, which allowed us to achieve a large sample size for both ^1^H-MRS and 18F-DOPA PET imaging. With a view toward developing predictive biomarkers for stratified clinical trials, we formally assessed the accuracy of these measures for classifying antipsychotic response and nonresponse. In our previous multicenter ^1^H-MRS study in first-episode psychosis, our a priori outcome variable was glutamate in ratio to creatine.^29^ In the current multicenter study, we were able to correct glutamate estimates for voxel tissue composition (Glu_corr_, our primary outcome variable) by applying the same software (Gannet) to extract voxel tissue fractions in data acquired across different MRI systems. This has the advantage that potential influences of voxel creatine content (otherwise often used as an internal standard) are avoided (see supplementary Discussion).

Our study also has several limitations. It is not possible to establish the proportion of the NR group that would meet Treatment Response and Resistance In Psychosis (TRRIP) consensus requirements for “TRS” ^68^ as we did not include a prospective trial of antipsychotic medication or collect objective evidence of adherence. As we only collected clinical data at a single time-point, we did not establish the stability of R/NR status. These factors could have led to a less clinical separation between the R and NR groups and reduced our ability to observe differences in glutamate or dopamine measures. While the R and NR groups did not differ in duration of illness or current antipsychotic dose, the inclusion of patients who had been taking antipsychotic medication for some time may have influenced both 18F-DOPA *K*_i_^cer^^70^ and ^1^H-MRS glutamate^71^ values. The absence of a healthy control group means that we are unable to interpret ^1^H-MRS glutamate and 18F-DOPA *K*_i_^cer^ values in comparison to what may be expected in psychiatrically healthy individuals. Neither the ^1^H-MRS glutamate nor 18F-DOPA PET dopamine imaging measures specifically index neurotransmission. ^1^H-MRS estimates the total amount of intracellular glutamate in the voxel, including neurons as well as other cell types. 18F-DOPA is used to index presynaptic dopamine synthesis and storage capacity rather than dopamine release. Previous studies of glutamate in relation to antipsychotic response/nonresponse at a field strength of 3 tesla have detected differences in glutamate or Glx.^14,26–30^ Although glutamate values obtained with short TE PRESS at 3 tesla are routinely reported and published, glutamate can be difficult to reliably quantify without specialized sequences. Despite the fitting methods, the glutamate signal is likely to include some contamination from glutamine and macromolecules and this may vary across the site. As for other imaging modalities, there was between-scanner variation in both ^1^H-MRS and PET data, which will have reduced the sensitivity of our study. Although we did not detect significant site by group interactions, we cannot exclude the possibility that scanner variation impacted our results. Between-scanner variation is discussed further in the supplementary Discussion.

The results highlight the importance of considering age and sex effects in future studies of glutamate in schizophrenia. A lower level of ACC Glu_corr_ in older patients with schizophrenia is consistent with other reports.^59,72^ There is some evidence that age-related decline in ACC glutamate^58^ is greater in schizophrenia than in healthy aging,^60,72^ although other studies have reported similar rates of ACC glutamate decrease in patients and healthy volunteers.^59,73^ Our finding of higher ACC Glu_corr_ levels in male compared with female participants is less clear due to the relatively small number of female participants.

In conclusion, our findings support previous research linking increases in ACC glutamate to a poor antipsychotic response. However, the poor group discrimination suggests that glutamate ^1^H-MRS or ^18^F-DOPA measures alone cannot distinguish between antipsychotic responsive and nonresponsive groups after a mean of 5–6 years of illness. Multicenter, cross-platform ^1^H-MRS and PET studies are rare, and in future studies, sensitivity may be improved through greater harmonization. It is also possible that glutamatergic and dopaminergic markers may have more predictive power earlier in the course of the disorder before the potentially confounding effects of treatment and illness duration have taken effect. They may also have increased predictive power in combination with other factors that may associate with antipsychotic response, such as clinical and demographic measures,^11–13^ brain network connectivity,^74^ genetic factors,^75,76^ and blood measures.^77^ We plan to address these issues in future studies.

## Funding

This research was supported by the Medical Research Council (MRC) UK, Stratified Medicines Initiative, reference MR/L011794/1 ‘STRATA’. Research at the London site was supported by the Department of Health via the National Institute for Health Research (NIHR) Specialist Biomedical Research Center for Mental Health award to South London and Maudsley NHS Foundation Trust (SLaM) and the Institute of Psychiatry, Psychology and Neuroscience at King’s College London. The views expressed are those of the authors and not necessarily those of the NHS, the NIHR, or the Department of Health.

## Supplementary Material

sbaa128_suppl_Supplementary_Information

## Data Availability

At the time of submission, the data governance frameworks are being put in place to make a fully anonymized version of the data available to the wider research community via TranSMART data sharing platform: https://transmartfoundation.org/, which will be hosted at the MRC eMedLab: https://www.emedlab.ac.uk/. To apply for access to the data, please contact J.H.M. at james.maccabe@kcl.ac.uk.
