# Supplementary material for: Dopamine and Glutamate in Antipsychotic-Responsive Compared With Antipsychotic-Nonresponsive Psychosis: A Multicenter Positron Emission Tomography and Magnetic Resonance Spectroscopy Study (STRATA)
Source: Schizophr Bull. 2020 Sep 10;47(2):505–16. doi: 10.1093/schbul/sbaa128 (PMC7965076; doi:10.1093/schbul/sbaa128)
Supplement: sbaa128_suppl_Supplementary_Information [file sbaa128_suppl_supplementary_information.pdf]

Supplementary information

Dopamine and Glutamate in Antipsychotic-Responsive compared to Antipsychotic Non-Responsive Psychosis: A Multicentre Positron Emission Tomography and Magnetic Resonance Spectroscopy Study (STRATA).

Supplementary Methods

Proton Magnetic Resonance Spectroscopy (^{1}H-MRS).

Glutamate levels were measured using ^{1}H-MRS at all four sites. Data were acquired at 3 Tesla, on General Electric (GE) MR750 (General Electric Healthcare, Chicago, USA, KCL site), Philips Achieva (Philips Healthcare, The Netherlands, UoM site), Siemens Verio (UoE) or Siemens Prisma (CU) (Siemens Healthcare, Erlangen, Germany) magnetic resonance systems.

Phantom data

Prior to collection of *in vivo* patient data, each site acquired repeated *in vitro* ^{1}H-MRS phantom data and one 'pilot' scan in a healthy volunteer to check data quality. Phantoms consisted of round-bottom flasks containing a 300mL buffered solution of 0.05M N-acetyl aspartate (NAA) and 0.05M glutamate, titrated to pH 7.4 and prepared according to the LCModel manual.[1] To help ensure identical metabolite concentrations and MR relaxation times, a single batch of solution was prepared by the lead site, which was then divided into separate flasks and distributed to each site. Phantom solutions were stored refrigerated and brought to room temperature before imaging. Phantom ^{1}H-MRS spectra were acquired in a 20 x 20 x 20mm voxel positioned in the centre of the phantom solution using a similar PRESS acquisition to the *in vivo* data but with a reduced number of averages (TE = 35 msec; TR = 2000 msec; 32 averages). All sites obtained good quality spectra from phantoms as indicated by low linewidth, high SNR and low metabolite CRLB. Site differences were indicated in phantom data (Supplementary Table 5).

In vivo data.

Participants were positioned as comfortably as possible within the head-coil to minimize the likelihood of head motion. Soft padding was used to help them maintain this position. The total MRI scan duration was approximately 45 minutes. Prior to ^{1}H-MRS acquisition, whole brain 3D sagittal T1-weighted images were acquired, based on the Alzheimer's Disease Neuroimaging (ADNI) recommendations (ADNI-II/ADNI-GO,

http://adni.loni.usc.edu/methods/documents/mri-protocols/). T1-weighted images were automatically reformatted to provide axial, sagittal and coronal views. ^{1}H-MRS voxels were positioned on the axial plane and checked across all orientations. Non-rotated ^{1}H-MRS voxels were positioned in the ACC (20 x 20 x 20 mm^3, 16 mm superior to the anterior portion of the genu of the corpus callosum on the midline sagittal localiser),[2] and in the right striatum (caudate nucleus, 20 x 20 x 20 mm^3, with the lower end of the voxel located 3 mm dorsal to the anterior commissure, including the maximum amount of grey matter and minimum amount of cerebrospinal fluid)[3] (Supplementary Figures 1 and 2). ^{1}H-MRS spectra were acquired using Point RESolved Spectroscopy (PRESS, echo time = 35 ms; repetition time = 2000 ms; 128 averages, bandwidth/sample frequency ± 2500 Hz, number of complex points = 4096). On the GE systems data were acquired using the standard GE PROBE (PROton Brain Examination) sequence. Unsuppressed water spectra were acquired in the same voxel locations.

Spectra were analyzed in LCModel version 6.3-1L[1] using a standard LCModel basis set acquired using PRESS at 3 Tesla and a TE of 35 ms containing 16 metabolites (L-alanine, aspartate, creatine, phosphocreatine, GABA, glucose, glutamine, glutamate, glycerophosphocholine, glycine, myo-inositol, L-lactate, N-acetylaspartate, N-acetylaspartylglutamate, phosphocholine, taurine). All metabolite estimates were water-referenced. Example *in vivo* spectra at each site are provided in Supplementary Figure 3.

Gannet software (version 2.0, http://www.gabamrs.com/), running in Matlab (version 9.2.0, MathWorks, USA) with SPM-8 (revision 6313, http://www.fil.ion.ucl.ac.uk/spm/) was used to co-register the ^{1}H-MRS voxel to the corresponding T1-weighted image and determine the voxel grey matter (GM), white matter (WM) and CSF fractions from the segmented T1-weighted image. Metabolite values were corrected for voxel tissue content using the formula:

$$Mcorr = M*(WM + 1.21*GM + 1.55*CSF) / (WM + GM)$$

where M is the uncorrected metabolite concentration/ level, and WM, GM and CSF indicate the percentages of tissue content in the voxel.[4, 5] The terms in the numerator are the ratios of the water content for each tissue type to the water content of white matter, since by default voxels are assumed to be pure white matter in the LCModel analysis. Since a relatively short TE and long TR were used, no corrections were applied for metabolite and tissue water T_1 and T_2 relaxation times, except for assuming T_2 of tissue water = 80 ms.

^{18}F-DOPA Positron Emission Tomography (PET)

Striatal dopamine function was measured using ^{18}F-DOPA PET. The study acquired ^{18}F-DOPA PET scans at two sites (KCL and the UoM). Participants were asked to fast, avoid drinking (apart from water) from midnight on the night before the PET scan. Participants were asked not to smoke for 2 hours before the scan, and not to take illicit drugs in the three days prior to the scan. Urine samples were taken on the day of the scan to check for the presence of illicit drugs and to exclude pregnancy. To reduce the formation of radiolabeled ^{18}F-DOPA metabolites,[6] participants received carbidopa (150 mg) and entacapone (400 mg) orally 1 hour before ^{18}F-DOPA imaging.[7]

Data were acquired on a Siemens Biograph 6 HiRez PET/CT scanner (Siemens, Erlangen, Germany) (KCL site) or a Siemens Biograph TruePoint TrueV PET/CT scanner (Siemens, Erlangen, Germany) (UoM site) in three-dimensional mode. Head positioning was monitored with laser cross-hairs and head movement was minimized by a light head strap. A CT scan was acquired for attenuation correction. Thirty seconds after the start of PET image acquisition, approximately 150 MBq of ^{18}F-DOPA was administered by bolus intravenous injection. Emission data were acquired in list mode over 32 frames (8x15 seconds, 3x60 seconds, 5x120 seconds, 16x300 seconds) over the 95-minute period immediately post-injection.

Raw data were transferred to KCL for analysis. Head movement was corrected for by frame-by-frame realignment using mutual information image registration.[8, 9] An ^{18}F-DOPA template,[10] together with a striatal brain atlas,[11] and cerebellum[12] were normalized to each ^{18}F-DOPA PET summation image in Statistical Parametric Mapping version 12 (http//www.fil.ion.ucl.ac.uk/spm) running in Matlab 2015b (Mathworks Inc. Sherbon MA, USA). This process allows automatic placement of volumes of interest (VOI) on individual subject PET images. The rate constant for the uptake of ^{18}F-DOPA in the striatum (K_i^{cer} min^{-1}) was calculated using graphical analysis adapted for a reference tissue input function, using the cerebellum as the reference region.[13, 14] Our previous test-retest study in healthy volunteers shows this method has good reliability, with an overall striatal intraclass correlation coefficient of 0.84.[15] Example ^{18}F-DOPA PET K_i^{cer} images at each site are shown in Supplementary Figure 4.

To account for site effects, both ^{1}H-MRS metabolite estimates and ^{18}F-DOPA PET K_i^{cer} values were converted to Z scores for hypothesis testing. Z scores were calculated by subtracting the site mean from individual values, before dividing by the site standard deviation. Table 3 in the

main text and Supplement Table 4 present the non-converted values by site as well as the overall z-scores.

Results: Site effects

There were significant site effects for values relating to ^{1}H-MRS spectral quality (Supplementary Table 3) and metabolite concentration estimates across *in vivo* data (Supplementary Table 4). For spectral quality measures, ANOVA revealed significant effects of site on spectral line-width (FWHM; ACC $F_{3,78} = 6.66$; $P < 0.001$; striatum: $F_{3,78} = 9.28$; $P < 0.001$) and signal to noise ratio (SNR; ACC $F_{3,78} = 27.02$; $P < 0.001$; striatum $F_{3,78} = 8.45$; $P < 0.001$). CRLB for individual metabolite estimates showed significant effects of site for myoinositol and choline in the ACC, and all metabolites except myoinositol in the caudate ($P < 0.05$). Spectral quality measures did not differ significantly by Responder / Non-Responder group, and site by group interactions were also non-significant. Post hoc analysis indicated that FWHM and SNR values in both voxels were lowest at UoM < KCL=CU < UoE. Site effects in ^{1}H-MRS metabolite concentration estimates were generally attributed to higher values being obtained on the GE scanner at the KCL site. Site effects were also apparent for 18F-DOPA K_i^{cer} PET data (Main Manuscript, Table 3).

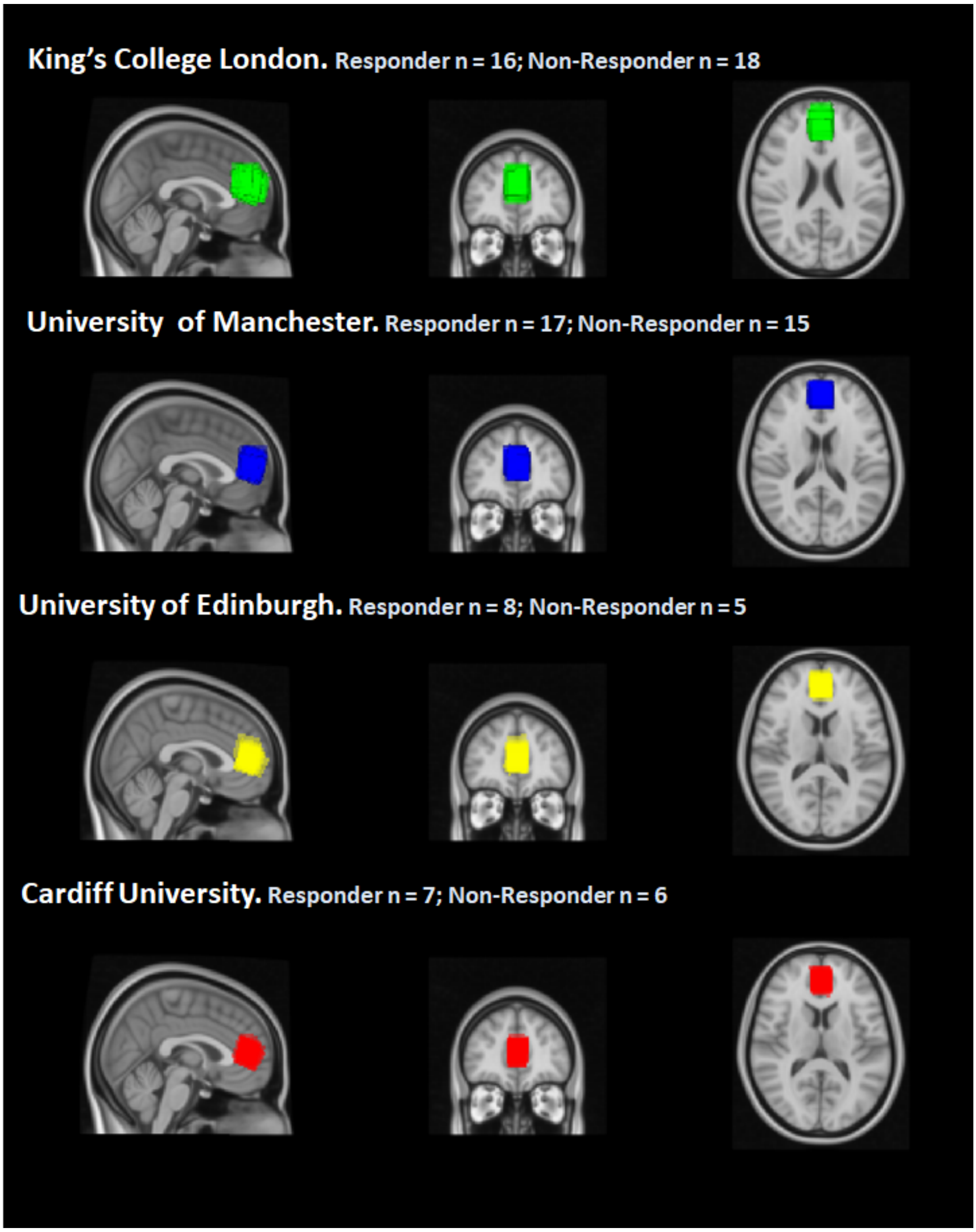

Supplementary Figure 1. ^{1}H-MRS voxel placement in the anterior cingulate cortex (ACC) at each study site. The images show overlaid voxels (each 20 x 20 x 20 mm^3) from each study participant. The number of participants in the Antipsychotic Responder and Antipsychotic Non-Responder groups at each site is provided.

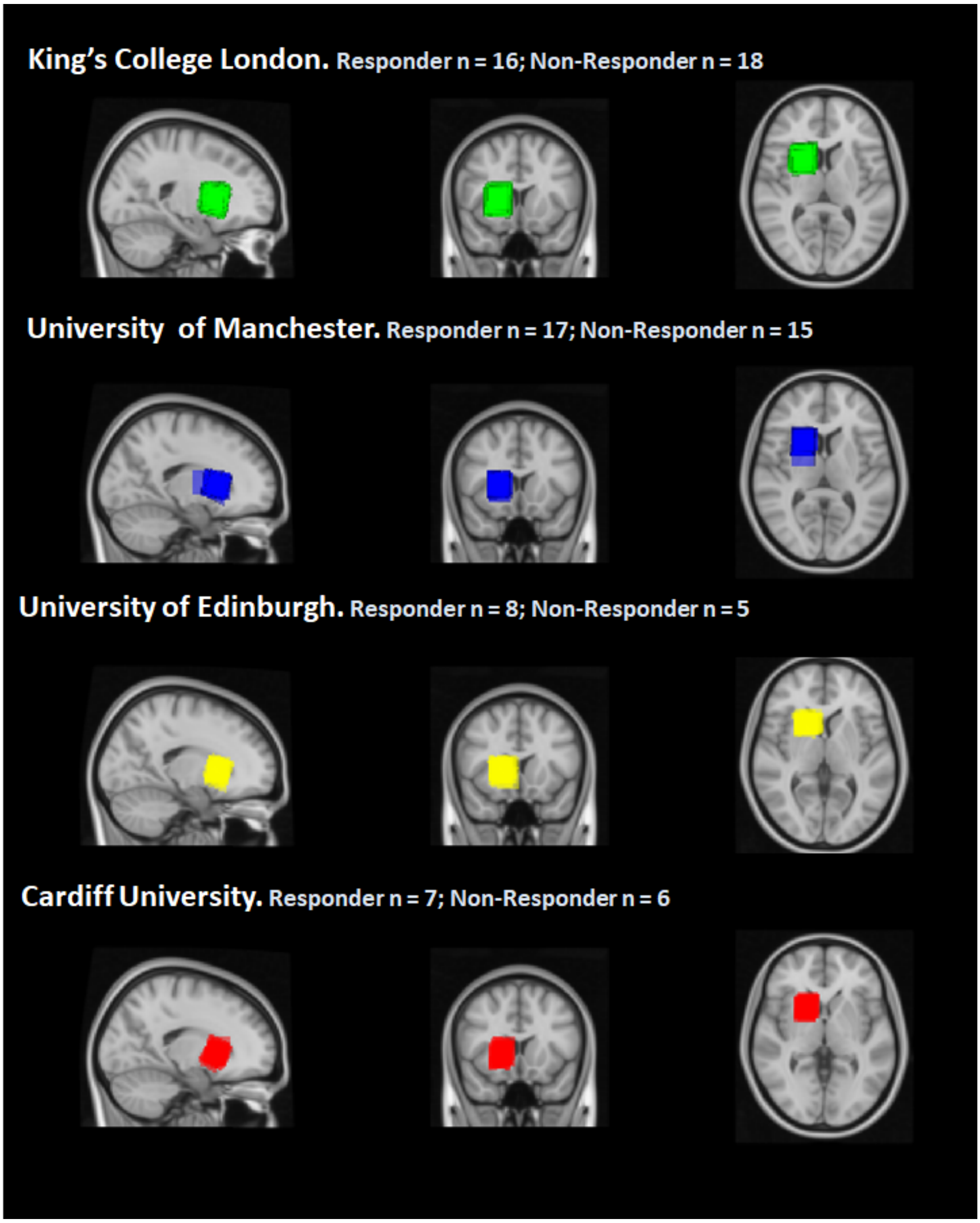

Supplementary Figure 2. ^{1}H-MRS voxel placement in the right striatum (caudate nucleus) at each study site. The images show overlaid voxels (each 20 x 20 x 20 mm^3) from each participant. The number of participants in the Antipsychotic Responder and Antipsychotic Non-Responder groups at each site is provided.

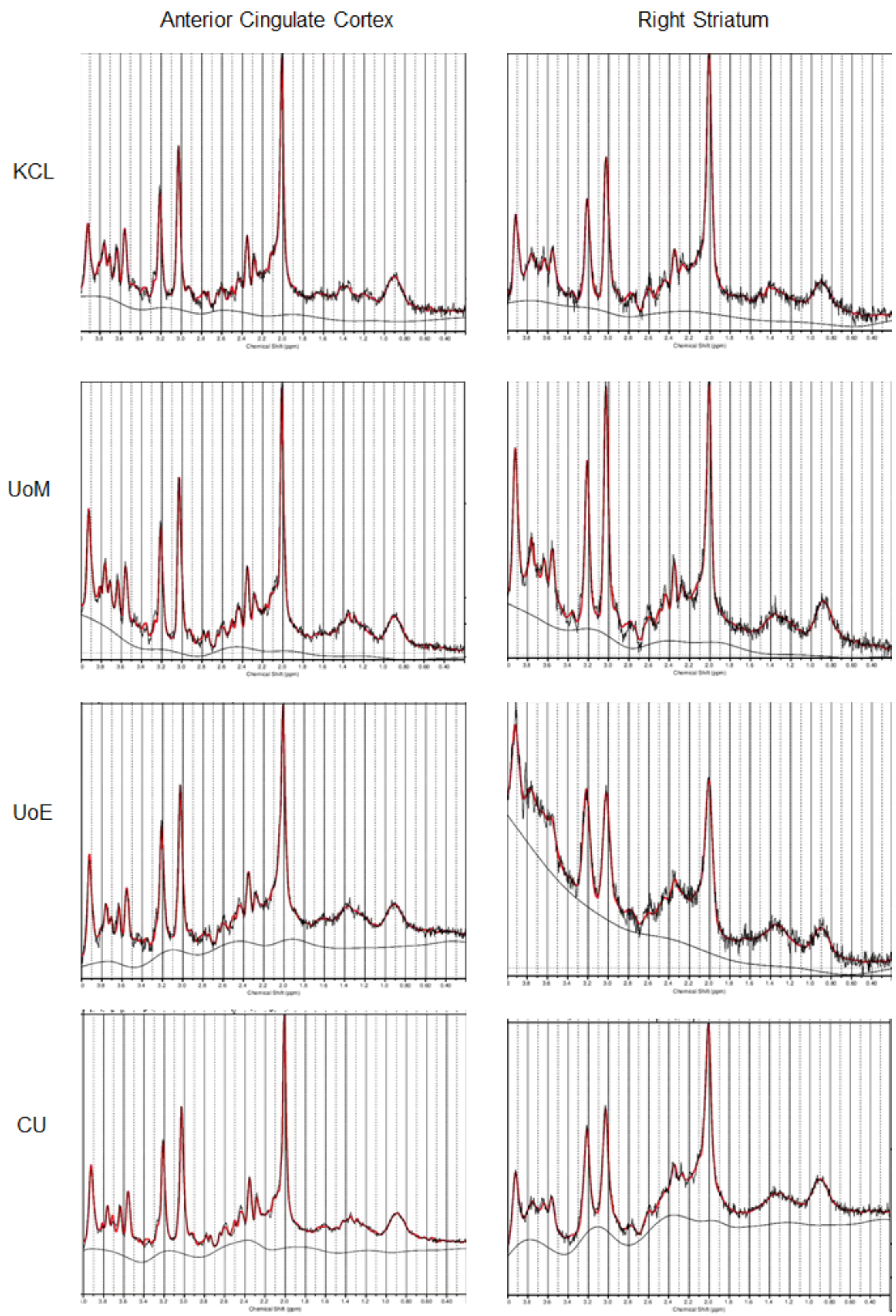

Supplementary Figure 3. Example ¹H-MRS spectra in the Anterior Cingulate Cortex and Right Striatum (caudate) at each study site. The figures show LCModel output of the fit in red, overlaid on the acquired spectrum (black). The estimated baseline is shown underneath in black.

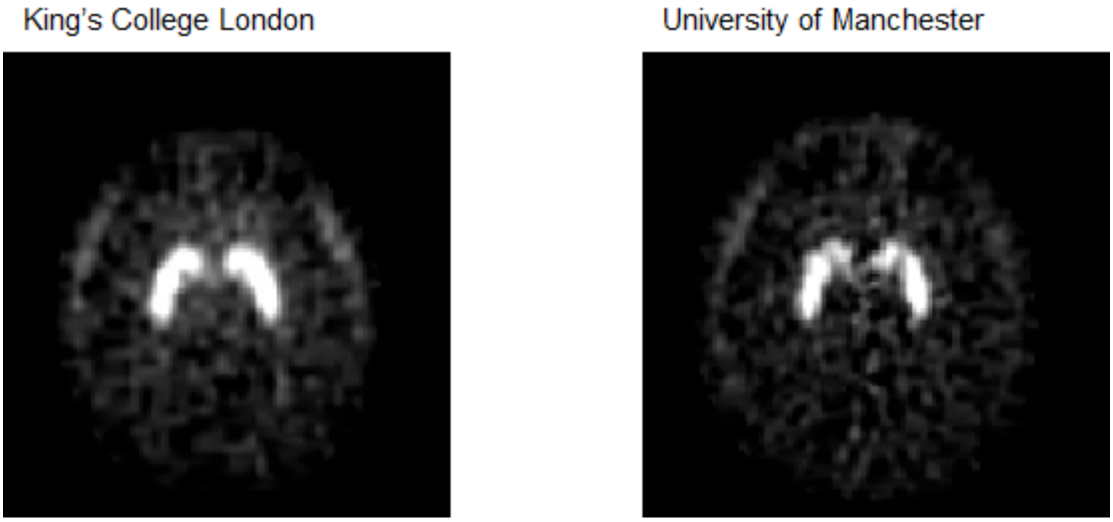

Supplementary Figure 4. Example ^{18}F-DOPA PET K_i^{cer} maps obtained at each study site. 18F-DOPA PET provides an index of presynaptic dopamine synthesis and storage capacity, with the most intense signal occurring bilaterally in the striatum.

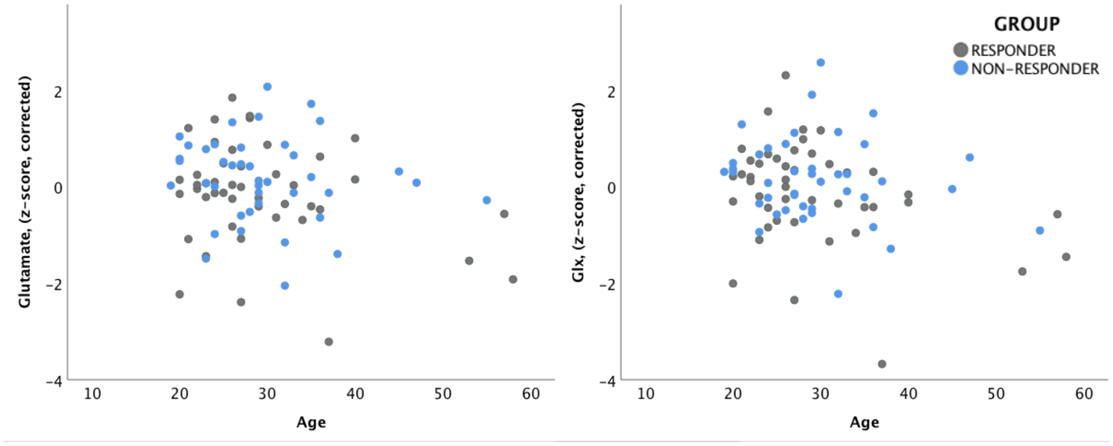

Supplementary Figure 5. Relationship between glutamate (left) and Glx (right) in the anterior cingulate cortex (ACC) and age in the Antipsychotic Responder and Antipsychotic Non-Responder groups. Glutamate and Glx are expressed as z-scores of the ^{1}H-MRS metabolite values, scaled to voxel tissue content (Glu_{corr} and Glx_{corr}). Across all participants, Glu_{corr} and Glx_{corr} significantly declined with age (N = 86; Glu_{corr} $r = -0.21$; $P = 0.05$; Glx_{corr} $r = -0.27$; $P = 0.01$).

	Site	Antipsychotic Responder	Antipsychotic Non-Responder
Sample size	KCL	16	18
	UoM	17	15
	UoE	8	5
	CU	7	6
	Overall	48	44
Age (years)	KCL	31 ± 11	31 ± 9
	UoM	27 ± 4	29 ± 7
	UoE	32 ± 10	29 ± 5
	CU	31 ± 15	24 ± 5
	Overall	29.9 ± 9.8	28.9 ± 7.5
Sex Male/Female	KCL	13/3	15/3
	UoM	15/2	13/2
	UoE	8/0	3/2
	CU	5/2	5/1
	Overall	41/7	36/8
CPZE mg/day	KCL	373.4 ± 207.2	483.6 ± 274.8
	UoM	406.2 ± 214.5	678.0 ± 511.5
	UoE	590.5 ± 263.4	267.2 ± 245.3
	CU	409.6 ± 318.0	394.2 ± 132.4
	Overall	426.5 ± 241.5	515.9 ± 379.6
Age onset	KCL	25 ± 4	24 ± 5
	UoM	23 ± 6	24 ± 9
	UoE	28 ± 7	23 ± 3
	CU	24 ± 12	21 ± 6
	Overall	24.6 ± 6.8	23.8 ± 6.4
Duration of illness	KCL	6 ± 11	6 ± 6
	UoM	4 ± 3	5 ± 4
	UoE	4 ± 4	6 ± 5
	CU	7 ± 11	3 ± 2
	Overall	5.1 ± 7.9	5.1 ± 5.1
PANSS positive	KCL	12 ± 3	23 ± 3
	UoM	13 ± 4	22 ± 3
	UoE	10 ± 3	23 ± 3
	CU	12 ± 2	23 ± 3
	Overall	12.0 ± 3.1	22.5 ± 3.5
PANSS negative	KCL	16 ± 3	24 ± 4
	UoM	12 ± 2	21 ± 5
	UoE	14 ± 5	18 ± 2
	CU	12 ± 2	16 ± 3
	Overall	13.5 ± 3.3	20.9 ± 4.8
PANSS general	KCL	28 ± 2	44 ± 4
	UoM	26 ± 4	41 ± 5
	UoE	27 ± 5	43 ± 8
	CU	29 ± 3	48 ± 4
	Overall	27.2 ± 4.3	43.3 ± 5.3
PANSS total	KCL	56 ± 2	91 ± 8
	UoM	51 ± 7	83 ± 10
	UoE	52 ± 7	84 ± 8
	CU	53 ± 5	86 ± 6
	Overall	52.7 ± 6.7	86.7 ± 8.8

Supplementary Table 1: Demographic and clinical characteristics of the ^{1}H-MRS sample at each study site. Data are expressed as mean ± standard deviation unless otherwise specified. CPZE: Chlorpromazine equivalent dose; PANSS: positive and negative syndrome scale.

	Site	Antipsychotic Responder	Antipsychotic Non-Responder
Sample size	KCL	11	16
	UoM	14	13
	Overall	25	29
Age (years)	KCL	33 ± 13	31 ± 9
	UoM	28 ± 5	29 ± 7
	Overall	29.8 ± 9.6	30.0 ± 8.3
Sex Male/Female	KCL	13/3	15/3
	UoM	15/2	13/2
	Overall	21/4	24/5
CPZE mg/day	KCL	353.7 ± 235.5	431.6 ± 244.1
	UoM	445.0 ± 215.7	713.1 ± 539.8
	Overall	404.8 ± 224.6	557.8 ± 420.9
Age onset	KCL	24 ± 4	24 ± 5
	UoM	23 ± 6	24 ± 9
	Overall	23.4 ± 5.5	24.1 ± 6.9
Duration of illness	KCL	8 ± 13	7 ± 7
	UoM	4 ± 4	5 ± 4
	Overall	5.9 ± 9.1	5.9 ± 5.7
PANSS positive	KCL	13 ± 2	24 ± 4
	UoM	13 ± 4	21 ± 4
	Overall	12.8 ± 3.2	22.0 ± 3.7
PANSS negative	KCL	15 ± 3	24 ± 4
	UoM	12 ± 2	21 ± 5
	Overall	13.3 ± 3.1	22.3 ± 4.4
PANSS general	KCL	28 ± 2	45 ± 4
	UoM	26 ± 3	40 ± 5
	Overall	27.0 ± 2.9	42.6 ± 5.1
PANSS total	KCL	56 ± 2	91 ± 9
	UoM	51 ± 6	82 ± 9
	Overall	53.1 ± 5.5	86.9 ± 9.5

Supplementary Table 2: Demographic and clinical characteristics of the ^{18}F-DOPA PET sample at each study site. Data are expressed as mean ± standard deviation unless otherwise specified. CPZE: Chlorpromazine equivalent dose; PANSS: positive and negative syndrome scale.

Anterior cingulate cortex			
	Site	Antipsychotic Responder	Antipsychotic Non-Responder
FWHM	KCL	0.039 ± 0.005 (16)	0.041 ± 0.009 (18)
	UoM	0.035 ± 0.005 (17)	0.037 ± 0.006 (15)
	UoE	0.049 ± 0.011 (7)	0.045 ± 0.008 (5)
	CU	0.044 ± 0.015 (5)	0.042 ± 0.010 (3)
SNR	KCL	26.3 ± 4.3 (16)	25.8 ± 2.9 (18)
	UoM	23.1 ± 4.7 (17)	23.3 ± 4.3 (15)
	UoE	24.3 ± 3.5 (7)	29.8 ± 2.6 (5)
	CU	36.8 ± 5.9 (5)	39.0 ± 1.0 (3)
GM	KCL	0.63 ± 0.06 (16)	0.62 ± 0.06 (18)
	UoM	0.65 ± 0.02 (17)	0.64 ± 0.04 (15)
	UoE	0.61 ± 0.05 (7)	0.62 ± 0.03 (5)
	CU	0.65 ± 0.06 (5)	0.65 ± 0.04 (3)
WM	KCL	0.11 ± 0.04 (16)	0.10 ± 0.04 (18)
	UoM	0.10 ± 0.05 (17)	0.08 ± 0.03 (15)
	UoE	0.09 ± 0.04 (7)	0.12 ± 0.03 (5)
	CU	0.08 ± 0.02 (5)	0.10 ± 0.01 (3)
CSF	KCL	0.27 ± 0.09 (16)	0.28 ± 0.09 (18)
	UoM	0.25 ± 0.06 (17)	0.28 ± 0.04 (15)
	UoE	0.30 ± 0.08 (7)	0.26 ± 0.05 (5)
	CU	0.27 ± 0.07 (5)	0.25 ± 0.02 (3)
Glu CRLB	KCL	5.81 ± 0.91 (16)	5.72 ± 0.67 (18)
	UoM	5.76 ± 1.03 (17)	6.00 ± 0.76 (15)
	UoE	6.71 ± 0.76 (7)	5.40 ± 0.55 (5)
	CU	5.80 ± 0.84 (5)	5.00 ± 1.00 (3)
Gln CRLB	KCL	15.67 ± 2.69 (9)	16.30 ± 2.41 (10)
	UoM	14.94 ± 2.17 (16)	15.77 ±1.83 (13)
	UoE	16.33 ± 2.07 (6)	14.20 ± 2.77 (5)
	CU	17.67 ± 1.15 (3)	15.67 ± 2.89 (3)
Glx CRLB	KCL	6.37 ± 1.45 (16)	6.22 ± 0.88 (18)
	UoM	5.71 ± 0.99 (17)	5.93 ± 0.70 (15)
	UoE	6.71 ± 0.49 (7)	5.60 ± 0.55 (5)
	CU	6.40 ± 1.14 (5)	5.67 ± 0.57 (3)
NAA CRLB	KCL	2.63 ± 0.72 (16)	2.67 ± 0.49 (18)
	UoM	2.59 ± 0.51 (17)	2.67 ± 0.62 (15)
	UoE	3.00 ± 0.82 (7)	2.20 ± 0.45 (5)
	CU	2.40 ± 0.89 (5)	2.33 ± 0.58 (3)
MI CRLB	KCL	5.06 ± 0.68 (16)	5.00 ± 0.49 (18)
	UoM	4.94 ± 0.66 (17)	4.73 ± 0.59 (15)
	UoE	5.43 ± 0.53 (7)	4.20 ± 0.45 (5)
	CU	4.40 ± 0.55 (5)	4.00 ± 0.00 (3)
Cho CRLB	KCL	2.94 ± 0.44 (16)	3.00 ± 0.00 (18)
	UoM	3.00 ± 0.35 (17)	3.07 ± 0.26 (15)
	UoE	3.29 ± 0.49 (7)	2.80 ± 0.45 (5)
	CU	2.60 ± 0.55 (5)	2.67 ± 0.58 (3)
Cr CRLB	KCL	2.31 ± 0.48 (16)	2.33 ± 0.49 (18)
	UoM	2.18 ± 0.39 (17)	2.40 ± 0.51 (15)
	UoE	2.57 ± 0.53 (7)	2.00 ± 0.00 (5)
	CU	2.20 ± 0.45 (5)	2.00 ± 0.00 (3)

Right Striatum			
	Site	Antipsychotic Responder	Antipsychotic Non-Responder
FWHM	KCL	0.066 ± 0.010 (16)	0.065 ± 0.010 (18)
	UoM	0.056 ± 0.010 (17)	0.058 ± 0.010 (15)
	UoE	0.086 ± 0.014 (6)	0.077 ± 0.017 (4)
	CU	0.074 ± 0.031 (6)	0.069 ± 0.021 (4)
SNR	KCL	19.2 ± 3.7 (16)	19.7 ± 2.5 (18)
	UoM	16.8 ± 2.7 (17)	17.4 ± 2.9 (15)
	UoE	12.8 ± 4.7 (6)	14.0 ± 2.2 (4)
	CU	16.5 ± 7.6 (6)	13.8 ± 5.7 (4)
GM	KCL	0.39 ± 0.09 (16)	0.42 ± 0.07 (18)
	UoM	0.43 ± 0.04 (17)	0.40 ± 0.06 (15)
	UoE	0.47 ± 0.06 (6)	0.48 ± 0.08 (4)
	CU	0.46 ± 0.04 (6)	0.62 ± 0.08 (4)
WM	KCL	0.59 ± 0.09 (16)	0.53 ± 0.14 (18)
	UoM	0.52 ± 0.05 (17)	0.56 ± 0.07 (15)
	UoE	0.40 ± 0.11 (6)	0.46 ± 0.07 (4)
	CU	0.47 ±0.05 (6)	0.35 ± 0.05 (4)
CSF	KCL	0.01 ± 0.02 (16)	0.05 ± 0.14 (18)
	UoM	0.05 ± 0.04 (17)	0.04 ± 0.03 (15)
	UoE	0.13 ± 0.12 (6)	0.06 ± 0.06 (4)
	CU	0.07 ± 0.05 (6)	0.03 ± 0.03 (4)
Glu CRLB	KCL	7.63 ± 1.63 (16)	7.67 ± 1.64 (18)
	UoM	7.88 ± 1.17 (17)	7.40 ± 0.99 (15)
	UoE	8.40 ± 1.82 (5)	9.25 ± 2.22 (4)
	CU	7.80 ± 1.30 (5)	11.00 ± 5.35 (4)
Gln CRLB	KCL	17.50 ± 2.08 (4)	17.50 ± 3.54 (2)
	UoM	14.75 ± 2.41 (12)	16.09 ± 2.55 (11)
	UoE	14.00 ± 4.08 (4)	16.67 ± 3.51 (3)
	CU	19.00 (1)	17.00 ± 2.83 (2)
Glx CRLB	KCL	9.00 ± 3.33 (16)	9.28 ± 2.05 (18)
	UoM	7.76 ± 1.48 (17)	7.27 ± 1.16 (15)
	UoE	7.80 ± 1.30 (5)	9.25 ± 3.10 (4)
	CU	10.00 ± 2.00 (5)	8.33 ± 3.51 (3)
NAA CRLB	KCL	3.00 ± 0.82 (16)	3.11 ± 0.68 (18)
	UoM	2.88 ± 0.49 (17)	3.13 ± 0.52 (15)
	UoE	4.83 ± 1.94 (6)	4.50 ± 1.00 (4)
	CU	6.67 ± 5.57 (6)	3.75 ± 0.96 (4)
MI CRLB	KCL	8.50 ± 1.86 (16)	9.22 ± 3.02 (18)
	UoM	8.18 ± 3.36 (17)	7.47 ± 1.96 (15)
	UoE	9.00 ± 2.00 (5)	8.00 ± 2.94 (4)
	CU	8.83 ± 4.54 (6)	10.25 ± 2.06 (4)
Cho CRLB	KCL	3.31 ± 0.60 (16)	3.56 ± 1.20 (18)
	UoM	2.94 ± 0.43 (17)	3.13 ± 0.35 (15)
	UoE	4.33 ± 2.16 (6)	4.00 ± 0.82 (4)
	CU	5.80 ± 5.72 (5)	4.00 ± 1.41 (4)
Cr CRLB	KCL	2.88 ± 0.50 (16)	3.06 ± 0.42 (18)
	UoM	2.41 ± 0.51 (17)	2.40 ± 0.51 (15)
	UoE	4.17 ± 2.04 (6)	4.00 ± 0.82 (4)
	CU	5.40 ± 5.94 (5)	3.50 ± 1.00 (4)

Supplementary Table 3: ^{1}H-MRS data quality measures by study site in the Antipsychotic Responder and Antipsychotic Non-Responder groups.

Data are expressed as mean ± standard deviation (n). FWHM: full width at half maximum linewidth (ppm); SNR: signal to noise ratio; GM: voxel grey matter (%); WM: voxel white matter (%); CSF: voxel

cerebrospinal fluid (%); CRLB: Cramer Rao Lower Bounds (%); Glu: glutamate; Gln: glutamine; Glx: glutamate plus glutamine; NAA: N-acetylaspartate; MI: myo-inositol; Cho: choline; Cr: creatine.

Anterior cingulate cortex ^{1}H-MRS metabolites			
	Site	Antipsychotic Responder	Antipsychotic Non-Responder
Glu	KCL	19.39 ± 3.56 (16)	20.29 ± 2.63 (18)
	UoM	13.74 ± 1.75 (17)	14.28 ± 1.59 (15)
	UoE	12.57 ± 1.18 (7)	13.33 ± 0.90 (5)
	CU	11.65 ± 1.68 (5)	11.57 ± 2.22 (3)
	Overall	-0.15 ± 1.05 (45)	0.17 ± 0.88 (41)
Gln	KCL	7.99 ± 2.23 (9)	8.39 ± 1.73 (10)
	UoM	6.11 ± 0.90 (16)	6.04 ± 1.10 (13)
	UoE	6.24 ± 1.38 (6)	6.29 ± 1.20 (5)
	CU	3.71 ± 0.62 (3)	3.99 ± 0.38 (3)
	Overall	-0.61 ± 2.48 (34)	-0.31 ± 2.33 (31)
Glx	KCL	25.86 ± 5.28 (16)	26.85 ± 4.28 (18)
	UoM	19.67 ± 2.56 (17)	20.17 ± 1.87 (15)
	UoE	18.59 ± 1.61 (7)	19.62 ± 1.87 (5)
	CU	14.82 ± 2.15 (5)	15.56 ± 2.14 (3)
	Overall	-0.13 ± 1.06 (45)	0.15 ± 0.88 (41)
NAA	KCL	16.94 ± 3.10 (16)	17.25 ± 2.22 (18)
	UoM	11.32 ± 1.37 (17)	11.71 ± 0.98 (15)
	UoE	10.96 ± 2.00 (7)	12.56 ± 0.86 (5)
	CU	11.11 ± 2.11 (5)	10.53 ± 2.56 (3)
	Overall	-0.13 ± 1.1 (45)	0.14 ± 0.81 (41)
MI	KCL	10.17 ± 2.15 (16)	10.25 ± 1.15 (18)
	UoM	6.91 ± 0.79 (17)	7.64 ± 0.89 (15)
	UoE	7.00 ± 1.15 (7)	7.78 ± 0.56 (5)
	CU	7.08 ± 1.41 (5)	7.41 ± 1.35 (3)
	Overall	-0,21 ± 1.08 (45)	0.23 ± 0.82 (41)
Cho	KCL	3.36 ± 0.75 (16)	3.00 ± 0.00 (18)
	UoM	2.35 ± 0.32 (17)	3.07 ± 0.26 (15)
	UoE	2.26 ± 0.47 (7)	2.80 ± 0.45 (5)
	CU	2.27 ± 0.50 (5)	2.11 ± 0.31 (3)
	Overall	-0.06 ± 1.09 (45)	0.70 ± 0.86 (41)
Cr	KCL	12.18 ± 2.15 (16)	12.66 ± 1.51 (18)
	UoM	9.83 ± 0.99 (17)	10.10 ± 1.04 (15)
	UoE	8.83 ± 1.38 (7)	9.60 ± 0.79 (5)
	CU	8.99 ± 1.46 (5)	8.49 ± 1.24 (3)
	Overall	-0.11 ± 1.06 (45)	0.15 ± 0.87 (41)
**Right Striatum ^{1}H-MRS metabolites**			
	Site	Antipsychotic Responder	Antipsychotic Non-Responder
Glu	KCL	10.78 ± 1.47 (16)	11.07 ± 1.62 (18)
	UoM	8.35 ± 1.12 (17)	7.89 ± 1.01 (15)
	UoE	8.67 ± 1.13 (5)	7.41 ± 1.21 (4)
	CU	9.00 ± 3.15 (4)	8.58 ± 1.43 (4)
	Overall	0.10 ± 1.00 (42)	-0.11 ± 0.96 (41)
Gln	KCL	5.68 ± 0.55 (4)	5.88 ± 0.34 (1)
	UoM	4.95 ± 0.68 (12)	5.03 ± 1.88 (11)
	UoE	9.26 ± 4.94 (4)	6.27 ± 1.07 (3)
	CU	4.67 (1)	5.96 ± 2.06 (2)
	Overall	-0.15 ± 1.04 (21)	0.20 ± 1.04 (17)
Glx	KCL	15.08 ± 2.65 (16)	14.27 ± 3.15 (18)
	UoM	13.34 ± 1.81 (17)	12.89 ± 2.33 (15)
	UoE	16.69 ± 5.84 (5)	12.70 ± 3.20 (4)

	CU	11.75 ± 3.53 (4)	14.10 ± 3.41 (3)
	Overall	0.13 ± 0.91 (42)	-0.13 ± 1.04 (40)
NAA	KCL	11.26 ± 1.41 (16)	11.33 ± 0.80 (18)
	UoM	8.02 ± 0.64 (17)	7.55 ± 0.59 (15)
	UoE	8.56 ± 0.73(6)	7.40 ± 1.23 (4)
	CU	5.94 ± 2.35 (5)	8.83 ± 0.58 (4)
	Overall	0.11 ± 1.08 (44)	-0.11 ± 0.86 (41)
MI	KCL	4.19 ± 0.71 (16)	4.27 ± 1.56 (18)
	UoM	3.46 ± 0.78 (17)	3.69 ± 0.52 (15)
	UoE	3.86 ± 1.10 (3)	3.97 ± 1.58 (4)
	CU	5.65 ± 5.94 (5)	3.88 ± 1.21 (4)
	Overall	-0.02 ± 0.95 (41)	-0.04 ± 1.04 (41)
Cho	KCL	2.28 ± 0.28 (16)	2.15 ± 0.26 (18)
	UoM	1.92 ± 0.26 (17)	1.87 ± 0.17 (15)
	UoE	2.24 ± 0.43 (6)	1.82 ± 0.29 (4)
	CU	1.37 ± 0.59 (5)	2.00 ± 0.11 (4)
	Overall	0.13 ± 1.08 (44)	-0.14 ± 0.85 (41)
Cr	KCL	8.79 ± 0.89 (16)	8.69 ± 0.85 (18)
	UoM	7.85 ± 0.64 (17)	7.60 ± 0.56 (15)
	UoE	6.83 ± 1.16 (6)	5.73 ± 1.06 (4)
	CU	4.96 ± 2.67 (4)	7.14 ± 0.45 (4)
	Overall	0.10 ± 1.04 (43)	-0.10 ± 0.92 (41)

Supplementary Table 4: ^{1}H-MRS metabolite levels by study site in the Antipsychotic Responder and Antipsychotic Non-Responder groups.
All values are corrected for voxel tissue content (Mcorr). Data are expressed as mean ± standard deviation (n), by site, and overall as Z score. Glu: glutamate; Gln: glutamine; Glx: glutamate plus glutamine; NAA: N-acetylaspartate; MI: myo-inositol; Cho: choline; Cr: creatine.

	KCL	UoM	UoE	CU
Number of repeated measurements	7	5	4	4
Time period (days)	4	4	1	2
FWHM	0.025 ± 0.00	0.026 ± 0.005	0.056 ± 0.016	0.055 ± 0.04
SNR	52.71 ± 8.12	68.20 ± 1.79	54.75 ± 2.06	64.00 ± 6.48
NAA CRLB	2.29 ± 0.76	1.00 ± 0.00	1.00 ± 0.00	1.00 ± 0.00
Glu CRLB	3.43 ± 0.53	2.00 ± 0.00	2.75 ± 0.50	2.00 ± 0.00
NAA	49.89 ± 2.66	35.17 ± 0.98	35.30 ± 0.24	36.69 ± 2.37
Glu	47.24 ± 2.53	33.32 ± 1.08	32.21 ± 1.78	34.55 ± 1.03

Supplementary Table 5: ^{1}H-MRS phantom data by study site. Data are expressed as mean ± standard deviation. FWHM: full width at half maximum linewidth (ppm); SNR: signal to noise ratio; CRLB: Cramer Rao Lower Bounds (%); Glu: glutamate; NAA: N-acetylaspartate.

Supplementary Discussion

As with many other imaging modalities, both PET and ^{1}H-MRS suffer from between-scanner variation that impacts on comparisons of images across imaging sites, scanners and over time. There are several factors that are responsible for this variability. Some of these will be related directly to patient preparation and execution of the PET or MRI examination while others relate technical factors.

In our ^{1}H-MRS data, although we took steps to harmonize the acquisitions, by using the same water suppression method, echo time, bandwidth, centre frequency and number of data points, site effects were present in spectral signal to noise ratio (SNR), line-width (full width at half maximum, FWHM), metabolite fit (CRLB) and in metabolite concentration estimates. SNR and FWHM are directly affected both by the details of the scanner manufacturer's shimming software and the site-specific calibration of the scanner hardware, and as such some degree of inter-site variability is inevitable. Similar site effects in metabolite estimates have been observed in other multicentre ^{1}H-MRS studies acquiring data across scanners from different vendors.[16, 17] The higher values for metabolite estimates that were obtained on the GE scanner (KCL site) are consistent with previous reports of higher values on GE compared to Philips or Siemens scanners.[17] This effect is likely due to the use of outer volume saturation (OVS) using very selective saturation (VSS) pulses on the GE platform, which is not available on Philips or Siemens, leading to underestimation of the unsuppressed water peak.[18] In future multicenter studies, similarity of metabolite estimates across sites might be improved by avoiding VSS-OVS on GE platforms, although there are differences in other acquisition parameters (e.g. RF pulse shapes and timings) that may also need to be considered. Nonetheless, across sites and metabolites, CRLB were <10%. This indicates that site differences in spectral quality were sufficiently small, and that spectra were of sufficient quality, to allow good fit.

To standardize our analysis pipeline and avoid the potential of introducing additional inter-site variance when acquiring manufacturer or scanner-specific basis sets, we chose to use a single basis set to analyze all ^{1}H-MRS spectra. This is the recommended approach within LCModel,[1] in situations where the data to be analyzed match an existing basis set in terms of localization sequence (i.e. PRESS) and (to within a few percent), B0 field strength, and TE, as was the case in our study. At short echo times as applied in our protocol, TE1 varies for the different

manufacturers by ~ 1 ms, compared to the much larger variations that occur at longer TEs, which would affect the signals from strongly coupled spins. The limited number of other cross-platform multisite spectroscopy studies in psychiatry have also used single basis sets.[16, 19-21] Nonetheless Povazan *et al.*,[22] recently compared single voxel PRESS at TE=35ms in healthy volunteers across multiple sites and manufacturers, using manufacturer-specific basis sets, and found no effects of manufacturer for the ratio to creatine of Glx, NAA or mI. Site contributed more markedly than vendor to variance in metabolite estimates[22] although another multicenter study at 3 Tesla using a single manufacturer have found high inter-site reproducibility at a similar TE.[23] Overall, there are very few multisite / multi-manufacturer reproducibility studies at 3 Tesla and there currently a lack of consensus on the optimal parameters including the use a single or customized basis set when combining data. These will be topical issues as further multicenter ^{1}H-MRS consortia emerge.

Effects of PET scanner on K_i^{cer} have also been reported on previously.[24] This relates to the intrinsic sensitivity of the PET scanner as well as the reconstruction method and related parameters. As a result, PET images acquired in different sites can differ quite significantly in term on spatial image resolution, partial volume effects and noise content. Even with harmonization procedures to ensure comparable data quality are in place, residual differences in measured PET image may still be present.[25] In this project, despite having implemented a standardised PET acquisition protocol and analysis pipeline, the intrinsic differences of the KCL (Siemens Biograph 6 HiRez PET/CT) and Manchester (Siemens Biograph TruePoint TrueV PET/CT) scanners showed differences in PET data quality (Supplement Figure 4), which ultimately lead to differences in K_i estimates.

In addition to the main analysis investigating metabolite estimates corrected for voxel tissue composition (M_{corr}), for further comparison with previous reports we additionally analysed ACC glutamate and Glx in ratio to creatine. There were no significant effects of age or gender on ACC Glu/Cr or ACC Glx/Cr z-scores (P>0.05). The group effect on ACC Glu/Cr was not significant in an unadjusted model ($F_{1,84}$ = 0.31; P = 0.58), and was borderline significant for Glx/Cr ($F_{1,84}$ = 3.86; P = 0.05) Inspection of water-referenced creatine z-scores found no significant effects of age (n = 85; r = -0.12; P = 0.28), gender (T_{84} = 1.60; P = 0.11) nor group (T_{84} = 1.24; P = 0.22, Supplement Table 4). However, while creatine is often used as an internal reference in ^{1}H-MRS studies[26] and did not significantly differ between the patient groups in our study, there are also reports of creatine differences in schizophrenia,[27, 28] and as a ratio denominator, small group differences in creatine may also influence results.

References

1. Provencher SW. Estimation of metabolite concentrations from localized in vivo proton NMR spectra. *Magn ResonMed* 1993;30(6):672-679.
2. Egerton A, Brugger S, Raffin M, Barker GJ, Lythgoe DJ, McGuire PK, Stone JM. Anterior cingulate glutamate levels related to clinical status following treatment in first-episode schizophrenia. *Neuropsychopharmacology* 2012;37(11):2515-2521.
3. de la Fuente-Sandoval C, Leon-Ortiz P, Favila R, Stephano S, Mamo D, Ramirez-Bermudez J, Graff-Guerrero A. Higher levels of glutamate in the associative-striatum of subjects with prodromal symptoms of schizophrenia and patients with first-episode psychosis. *Neuropsychopharmacology* 2011;36(9):1781-1791.
4. Kreis R, Ernst T, Ross BD. Development of the human brain: in vivo quantification of metabolite and water content with proton magnetic resonance spectroscopy. *Magn Reson Med* Oct 1993;30(4):424-437.
5. Gasparovic C, Song T, Devier D, et al. Use of tissue water as a concentration reference for proton spectroscopic imaging. *Magn Reson Med* Jun 2006;55(6):1219-1226.
6. Cumming P, Leger GC, Kuwabara H, Gjedde A. Pharmacokinetics of plasma 6-[18F]fluoro-L-3,4-dihydroxyphenylalanine ([18F]Fdopa) in humans. *Journal of cerebral blood flow and metabolism : official journal of the International Society of Cerebral Blood Flow and Metabolism* 1993;13(4):668-675.
7. Sawle GV, Burn DJ, Morrish PK, Lammertsma AA, Snow BJ, Luthra S, Osman S, Brooks DJ. The effect of entacapone (OR-611) on brain [18F]-6-L-fluorodopa metabolism: implications for levodopa therapy of Parkinson's disease. *Neurology* 1994;44(7):1292-1297.
8. Studholme C, Hill DL, Hawkes DJ. Automated 3-D registration of MR and CT images of the head. *Med Image Anal* 1996;1(2):163-175.
9. Turkheimer FE, Brett M, Visvikis D, Cunningham VJ. Multiresolution analysis of emission tomography images in the wavelet domain. *J CerebBlood Flow Metab* 1999;19(11):1189-1208.
10. McGowan S, Lawrence AD, Sales T, Quested D, Grasby P. Presynaptic dopaminergic dysfunction in schizophrenia: a positron emission tomographic [18F]fluorodopa study. *Archives of general psychiatry* 2004;61(2):134-142.
11. Martinez D, Slifstein M, Broft A, et al. Imaging human mesolimbic dopamine transmission with positron emission tomography. Part II: amphetamine-induced dopamine release in the functional subdivisions of the striatum. *Journal of cerebral blood flow and metabolism : official journal of the International Society of Cerebral Blood Flow and Metabolism* 2003;23(3):285-300.
12. Bloomfield MA, Pepper F, Egerton A, et al. Dopamine function in cigarette smokers: an [(1)(8)F]-DOPA PET study. *Neuropsychopharmacology* Sep 2014;39(10):2397-2404.

13. Patlak CS, Blasberg RG. Graphical evaluation of blood-to-brain transfer constants from multiple-time uptake data. Generalizations. *Journal of cerebral blood flow and metabolism : official journal of the International Society of Cerebral Blood Flow and Metabolism* 1985;5(4):584-590.

14. Turkheimer FE, Aston JA, Asselin MC, Hinz R. Multi-resolution Bayesian regression in PET dynamic studies using wavelets. *Neuroimage* 2006;32(1):111-121.

15. Egerton A, Demjaha A, McGuire P, Mehta MA, Howes OD. The test-retest reliability of 18F-DOPA PET in assessing striatal and extrastriatal presynaptic dopaminergic function. *Neuroimage* 2010;50(2):524-531.

16. Egerton A, Broberg BV, Van Haren N, et al. Response to initial antipsychotic treatment in first episode psychosis is related to anterior cingulate glutamate levels: a multicentre (1)H-MRS study (OPTiMiSE). *Mol Psychiatry* Nov 2018;23(11):2145-2155.

17. Naaijen J, Zwiers MP, Amiri H, et al. Fronto-Striatal Glutamate in Autism Spectrum Disorder and Obsessive Compulsive Disorder. *Neuropsychopharmacology* Nov 2017;42(12):2466-2467.

18. Rotaru D, Tsivaka D, Lythgoe D. Effects of outer volume saturation RF pulses and chemical shift displacement on MRS data. *ISMRM 27th Annual Meeting*. Montreal, Canada; 2019.

19. Craig MC, Mulder LM, Zwiers MP, et al. Distinct associations between fronto-striatal glutamate concentrations and callous-unemotional traits and proactive aggression in disruptive behavior. *Cortex* Dec 2019;121:135-146.

20. Naaijen J, Zwiers MP, Amiri H, et al. Fronto-Striatal Glutamate in Autism Spectrum Disorder and Obsessive Compulsive Disorder. *Neuropsychopharmacology* Nov 2017;42(12):2456-2465.

21. Naaijen J, Zwiers MP, Forde NJ, et al. Striatal structure and its association with N-Acetylaspartate and glutamate in autism spectrum disorder and obsessive compulsive disorder. *Eur Neuropsychopharmacol* Jan 2018;28(1):118-129.

22. Povazan M, Mikkelsen M, Berrington A, et al. Comparison of Multivendor Single-Voxel MR Spectroscopy Data Acquired in Healthy Brain at 26 Sites. *Radiology* Apr 2020;295(1):171-180.

23. Graf C, MacMillan EL, Fu E, et al. Intra- and inter-site reproducibility of human brain single-voxel proton MRS at 3 T. *NMR Biomed* Jun 2019;32(6):e4083.

24. Egerton A, Chaddock CA, Winton-Brown TT, Bloomfield MA, Bhattacharyya S, Allen P, McGuire PK, Howes OD. Presynaptic striatal dopamine dysfunction in people at ultra-high risk for psychosis: findings in a second cohort. *Biol Psychiatry* Jul 15 2013;74(2):106-112.

25. Makris NE, Huisman MC, Kinahan PE, Lammertsma AA, Boellaard R. Evaluation of strategies towards harmonization of FDG PET/CT studies in multicentre trials: comparison of scanner validation phantoms and data analysis procedures. *Eur J Nucl Med Mol Imaging* Oct 2013;40(10):1507-1515.

26. Schwerk A, Alves FD, Pouwels PJ, van Amelsvoort T. Metabolic alterations associated with schizophrenia: a critical evaluation of proton magnetic resonance spectroscopy studies. *J Neurochem* Jan 2014;128(1):1-87.

27. Ongur D, Prescot AP, Jensen JE, Cohen BM, Renshaw PF. Creatine abnormalities in schizophrenia and bipolar disorder. *Psychiatry Res* Apr 30 2009;172(1):44-48.

28. Bustillo JR, Jones T, Chen H, et al. Glutamatergic and Neuronal Dysfunction in Gray and White Matter: A Spectroscopic Imaging Study in a Large Schizophrenia Sample. *Schizophr Bull* May 1 2017;43(3):611-619.
